# The optimal alternative for quantifying reference evapotranspiration in climatic sub-regions of Bangladesh

**DOI:** 10.1038/s41598-020-77183-y

**Published:** 2020-11-19

**Authors:** Roquia Salam, Abu Reza Md. Towfiqul Islam, Quoc Bao Pham, Majid Dehghani, Nadhir Al-Ansari, Nguyen Thi Thuy Linh

**Affiliations:** 1 Department of Disaster Management, Begum Rokeya University, Rangpur, 5400 Bangladesh; 2 Environmental Quality, Atmospheric Science and Climate Change Research Group, Ton Duc Thang University, Ho Chi Minh City, Vietnam; 3 Faculty of Environment and Labour Safety, Ton Duc Thang University, Ho Chi Minh City, Vietnam; 4 Civil Engineering Department, Vali-e-Asr University of Rafsanjan, Rafsanjan, Iran; 5 Department of Civil, Environmental and Natural Resources Engineering, Lulea University of Technology, 97187 Lulea, Sweden; 6 Institute of Research and Development, Duy Tan University, Danang, 550000 Vietnam; 7 Faculty of Environmental and Chemical Engineering, Duy Tan University, Danang, 550000 Vietnam

**Keywords:** Atmospheric science, Climate change, Hydrology

## Abstract

Reference evapotranspiration (ET_o_) is a basic element for hydrological designing and agricultural water resources management. The FAO56 recommended Penman–Monteith (FAO56-PM) formula recognized worldwide as the robust and standard model for calculating ET_o_. However, the use of the FAO56-PM model is restricted in some data-scarce regions like Bangladesh. Therefore, it is imperative to find an optimal alternative for estimating ET_o_ against FAO56-PM model. This study comprehensively compared the performance of 13 empirical models (Hargreaves–Samani, HargreavesM1, Hargreaves M2, Berti, WMO, Abtew, Irmak 1, Irmak 2, Makkink, Priestley-Taylor, Jensen–Haise, Tabari and Turc) by using statistical criteria for 38-years dataset from 1980 to 2017 in Bangladesh. The radiation-based model proposed by Abtew (ET_o,6_) was selected as an optimal alternative in all the sub-regions and whole Bangladesh against FAO56-PM model owing to its high accuracy, reliability in outlining substantial spatiotemporal variations of ET_o_, with very well linearly correlation with the FAO56-PM and the least errors. The importance degree analysis of 13 models based on the random forest (RF) also depicted that Abtew (ET_o,6_) is the most reliable and robust model for ET_o_ computation in different sub-regions. Validation of the optimal alternative produced the largest correlation coefficient of 0.989 between ET_o,s_ and ET_o,6_ and confirmed that Abtew (ET_o,6_) is the best suitable method for ET_o_ calculation in Bangladesh.

## Introduction

Evapotranspiration (ET) is a physical aerodynamics process in which water moves from liquid to gaseous stage, whereas bringing from the soil to the atmospheric surface^1^. It denotes to both evaporation from vegetation and soil fields and transpiration from plants. Two distinct processes (evaporation and transpiration) happen concurrently, and there is no alternative way of differentiating one from the other. ET is one of the basic elements of the water cycle, and its estimation is necessary to drought mitigation and management as well as other fields, including agro-meteorology, hydrology, climatology, and environmental studies^2–5^. For this reason, many drought indices such as Reconnaissance Drought Index (RDI)^6^, Standardized Precipitation Evapotranspiration Index (SPEI)^7^, and the Water Surplus Variability Index (WSVI)^8^ are based on the ET. Besides, two closely associated terms and concepts are potential evapotranspiration (ETp) and reference evapotranspiration (ET_o_) that estimate the atmospheric evaporation demand. ETp is defined as the rate of water transpired in a specific time by a crop, fully shading the ground, of constant height with adequate soil water setting in the outline^9,10^. On the other hand, ET_o_ is expressed as the ET rate from a reference crop surface, where the reference crop surface is a theoretical grass or alfalfa with accurate and recognized characteristics^1,11^. However, the definition of ET_o_ is more precise and specific than the ETp. The application of the terms ETp and ET_o_ have been puzzled for several decades. One ideal example is that Hargreaves and Samani^12^ used the term “ETp” whereas again Hargreaves and Samani^13^ applied the term “ET_o_”. Under the well-known background of global warming in recent decades, the term ET_o_ has been broadly applied in hydrology^14–16^, agronomy field^17–19^, irrigation engineering^4,20,21^ and meteorological field^22,23^. The application of ET_o_ is also used in the studies of crop water demand. Therefore, knowledge of ET_o_ is of great importance in agricultural water management, hydrological field, climate change, and irrigation practice^24,25^.

FAO recommended Penman–Monteith (FAO56-PM) method is the sole standard method for estimating ET_o_^1,20,26^. The main limitation of the FAO56-PM method is the difficulty in obtaining all necessary input data (air temperature, humidity, solar radiation, and wind speed). In such circumstances, simple equations or alternative methods are often used to estimate ET_o_^27^. The major advantages of an optimal empirical method are the simplicity, low cost, ease of application, and easy access to a few climatic input data measured in most of meteorological stations^28^. Thus, an optimal empirical model is vital for ET_o_ estimation in data-limited regions across the globe, including Bangladesh.

The selection of an optimal empirical model for calculating the ET_o_ is significant for agricultural water resources management, hydrological planning and irrigation designing^11,24,29^. In recent decades, a large number of empirical models have been developed to estimate the ET_o_, which have been widely reported in the literature (Table S1). These empirical models can be demarcated into five types mainly based on the data requirement: mass transfer-based^30,31^, temperature-based^11^, radiation-based^13,32–36^, combined-based^24,33–35^ and the pan evaporation-based models^37–39^ (Table S1). Earlier studies showed that the performance of various empirical models exhibited spatiotemporal variations, and most of these empirical models might be region-specific that enhanced the uncertainty problem in the identified spatiotemporal patterns. To solve this issue, local adjustment and validation of empirical models are required against the standard FAO56-PM model at various regions with various climatic contexts for accurate estimation of the ET_o_^24^. Most of the previous studies revealed that the performance of empirical models for estimating ET_o_ showed significant regional differences^40,41^. For instance, the calibrated adjusted Hargreaves model performed better than the calibrated Priestley-Taylor model for measuring ET_o_ in Serbia^42^. Quej et al.^43^ assessed the performance of the temperature-based ET_o_ models and found that the Hargreaves–Samani model exhibited the best performance in a tropical sub-humid climate. Krishna^44^ pointed out that the Turc model was an optimal alternative for the estimation of the ET_o_ under a humid subtropical climate, India. Li et al.^24^ reported that combination-based Valiantzas3 was the best model for estimating ET_o_ in the humid to sub-humid region, China. On the contrary, Pandey and Pandey^45^ found that the Hargreaves–Samani method had a larger overestimation than the standard FAO56-PM in humid areas of India. These contrasting outcomes can be attributed to variations in regional climate, and geography. However, whether empirical models influence the computation procedure of the ET_o_ against the FAO56-PM model remains uncertain. Therefore, it is crucial to conduct research appraising the performance of the empirical models in Bangladesh to determine an optimal alternative to ET_o_ and their changes shifted overtimes at the regional scale and differ spatially^14,15^.

Bangladesh, the vast deltaic plain, a low-lying subtropical humid climatic country, is positioned in Southeast Asia, has a total land area of 147,700 square km^46^ (Supplementary Figure S1). The country has a complex geomorphic setting and complicated hydrologic system which comprises various water bodies, wetlands, floodplain, flood basins, agricultural land, forest, and hilly regions. The elevations of most regions of the country varied from 1 to 60 m above the mean sea level, which forms generally low-lying areas from the east to the west, making a so-called “delta-shaped” landform^4^. Nevertheless, Bangladesh is not only faced this type of difficulty in obtaining long-term and complete climatic datasets but also this poor country experiences similar phenomena owing to naturals such as a complicated hydro-geographic and climatic setting and humankind e.g., low economic growth, lack of proper knowledge and technological hindering causes. Under this circumstance, for the ET_o_ appraisal of Bangladesh, an alternative empirical model depending on the limited climatic dataset is needed^47^. Therefore, it is of paramount importance to validate an appropriate alternative model which is easier in calculation procedure with fewer climatic variable requirements and good precision in comparison with the FAO56-PM model in various climatic sub-regions of Bangladesh. To the author's knowledge, so far, a systematic and thorough investigation for choosing an optimal alternative for estimating ET_o_ has not been conducted in Bangladesh till now, particularly at a monthly and regional scale, which in itself is the novelty of this study.

Based on the aforementioned research gaps, in this research, 13 widely employed empirical models selected for performance evaluation, including four temperature-based model (Hargreaves–Samani, Hargreaves M1, Hargreaves M2, and Berti), one mass transfer-based models (WMO) and eight radiation-based models (Makkink, Priestly–Taylor, Jensen–Haise, Abtew, Irmak1, Irmak 2, Turc, and Tabari), based on the extensive literature review of meteorological variables, climatic regional differences, and their universal applicability. Subsequently, this study seeks three hypotheses: first, the various empirical models will generate considerably various outcomes for estimating the ET_o_ at a monthly and regional scale; second, identifying the importance degree of empirical models that can indicate which model is outperformed against the FAO56-PM model at the regional scale and third, the simple linear regression can efficiently validate the 13 models against the FAO56-PM model in different sub-regions and whole Bangladesh. The specific objectives of this study are: (1) to analyze the spatiotemporal changes and the trends of ET_o_ in Bangladesh for the period of 1980–2017 at a monthly scale, (2) to compare the performances of 13 empirical models against the FAO56-PM model for ET_o_ estimation in climatic sub-regions of Bangladesh, (3) to choose an optimal alternative of the FAO56-PM ET_o_ model, which will be easier in ET_o_ quantification and apply few meteorological variables, (4) to identify the most outperformed empirical model against FAO56-PM model using the heuristic random forest method, and (5) to validate 13 empirical models using linear regression to opt an alternative empirical model against FAO56-PM model. The novelty of this research lies in employing 13 empirical models with a heuristic random forest model for the first time in Bangladesh that enables us to find an optimal alternative used for important environmental implication from the most reliable and outperformed equations for ET_o_ computation in different climatic sub-regions of Bangladesh.

## Data and methods

### Study area description and data sources

**Figure 1:**
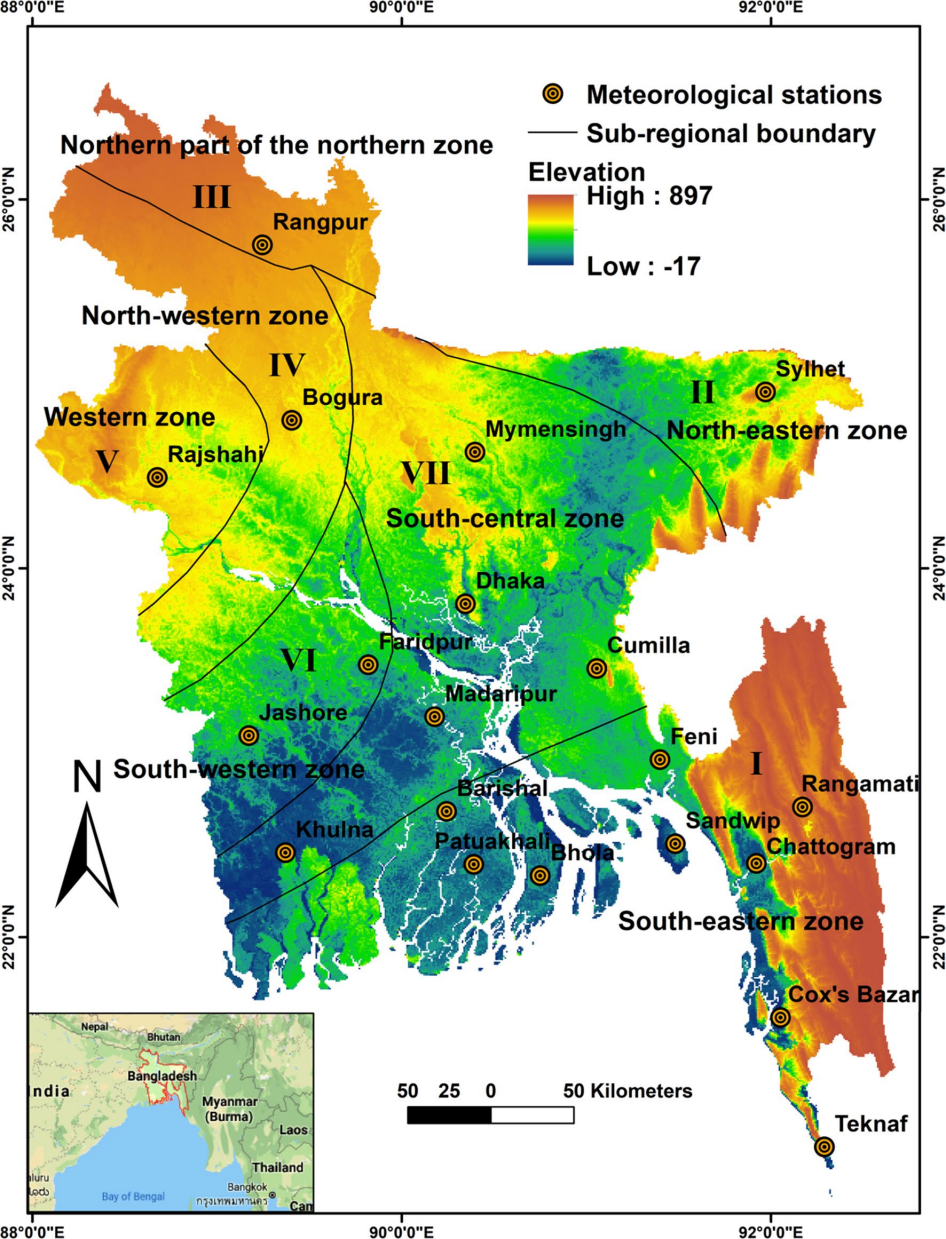
Map showing the geographical location of study area, prepared by ArcGis 10.5 (www.esri.com).

Bangladesh, situated in Southeast Asia, geographically it encompasses between 20° 30′ N and 26° 45′ N latitudes and 88° 0′ E to 92° 45′ E longitudes (Fig. 1). Banglapedia^48^ divided Bangladesh into seven climatic sub-regions based on climatology and geography as shown in the Supplementary Material of Figure S2. The seven sub-regions are (1) south-eastern zone; (2) north-eastern zone; (3) northern part of the northern zone; (4) north-western zone; (5) western zone; (6) south-western zone and (7) south-central zone. Bangladesh experiences a sub-tropical humid monsoon climate with seasonal differences^4^.

Western Bangladesh has usually become drier compared to other regions in Bangladesh^49^. Here, climatic variability is a regular scenario. Long-term daily average relative humidity, minimum temperature, maximum temperature, wind speed (at 2 m height), net radiation, evapotranspiration across the country are, respectively, 80%, 21.39 °C, 29.94 °C 1.32 ms^−1^, 10.44 MJm^−2^day^−1^, and 3.72 mm day^−1^.

Bangladesh Meteorological Department (BMD) runs only 43 meteorological stations across the country. The meteorological stations are unevenly distributed all over the country and most of the stations are located in the south-eastern regions. These meteorological stations are available for the climatic dataset, although some of the stations are newly established after the 1990s in Bangladesh and they do not have long-term data records (www.bmd.gov.bd). When more climatic variables are required, the dataset from a smaller number of stations was available. Due to these drawbacks, 20 stations were chosen for ET_o_ estimation over the 38 years from 1980 to 2017. These selected 20 stations embody the seven climatic sub-regions of the country. Daily minimum (T_min_) and maximum temperature (T_max_) (°C), mean relative humidity (Hr) (%), wind speed (Uz) (Knots) and sunshine hour (h day^−1^) datasets of 20 stations were sourced from the BMD. Net radiation (Rn) and wind speed at 2 m height (U2) cannot directly be measured by weather stations. Daily Rn and U2 were estimated using the procedures recommended by Allen et al.^1^ with the available meteorological datasets. A brief geographical and meteorological description of the selected stations is found in the Supplementary Material of Table S2. However, missing data in almost all the 20 stations was found. After the initial screening test, missing data of the 20 stations were less than 5% for the period of 1980–2017. Missing data for each station were filled by the existing records for the respective days from the adjacent neighbor stations. It is worthy to note that sunshine hour dataset in this study is continuous with no missing data. More details about the fill-up of missing meteorological datasets is given in the Supplementary Material (Table S3). The BMD follows the guideline of World Meteorological Organization (WMO) for weather dataset collection and record archiving. However, quality control of the dataset was primarily undertaken thoroughly by checking namely, positive values of parameters, for example, T_min_ is lower than T_max_, and humidity is less than 100%. The homogeneity tests of the dataset were conducted to exhibit any anomaly in the dataset^50^. All of the datasets were passed through the quality control by the staff of the BMD.

### FAO56 Penman–Monteith model (FAO56-PM model)

The FAO56-PM equation is used for estimating daily ET_o_ of this study. This model is well-known as the standard model for estimating ET_o_ across the whole world, which was proposed by Allen et al.^1^ The original form of FAO56-PM model is expressed by the following Eq. (1):

1 $${\text{ET}}_{{\text{o}}} = \frac{{0.408\Delta \left( {{\text{R}}_{{\text{n}}} - {\text{ G}}} \right) + {\upgamma }\frac{900}{{{\text{T}} + 273}}{\text{U}}_{2} \left( {{\text{e}}_{{\text{s}}} - {\text{e}}_{{\text{a}}} } \right)}}{{\Delta + {\upgamma }\left( {1{ } + { }0.34{\text{U}}_{2} } \right)}}$$

where, ET_o_ is the reference evapotranspiration (mm day^−1^),${\mathrm{R}}_{\mathrm{n}}$ isthe net radiation atcrop surface (MJm^−2^ day^−1^), Gis the soil heat flux density (MJ m^−2^ day^−1^), T is the average daily air temperature at 2 m height (°C), ${\mathrm{U}}_{2}$ is the wind speed at 2 m height (ms^−1^), ${\mathrm{e}}_{\mathrm{s}}$ is the saturation vapour pressure (kPa),${\mathrm{e}}_{\mathrm{a}}$ is the actual vapour pressure (kPa), ${\mathrm{e}}_{\mathrm{s}}-{\mathrm{e}}_{\mathrm{a}}$ is the saturation vapour pressure deficit (kPa), $\Delta$ is the slope of vapour pressure curve (kPa °C^−1^), γ is the psychrometric constant (kPa °C^−1^). Allen et al.^1^ recommended G = 0. The detailed procedures of ET_o_ estimation is found in FAO 56 paper^1^.

R_n_ is calculated by the Eqs. (2–11):

2 $${\text{R}}_{{\text{n}}} = {\text{R}}_{{{\text{ns}}}} - {\text{R}}_{{{\text{nl}}}}$$

3 $${\text{R}}_{{{\text{ns}}}} = ({1} - \alpha ){\text{R}}_{{\text{s}}}$$

4 $${\text{R}}_{{\text{s}}} = \left[ {{\text{a}}_{{\text{s}}} + {\text{b}}_{{\text{s}}} \frac{{\text{n}}}{{\text{N}}}} \right]{\text{ R}}_{{\text{a}}}$$

5 $${\text{Ra}} = \frac{{24{ }\left( {60} \right)}}{\pi } {\text{G}}_{{{\text{sc}}}} {\text{d}}_{{\text{r}}} [\omega_{{\text{s}}} {\text{sin}}(\varphi ){\text{sin}}(\delta ) + {\text{cos}}(\varphi ){\text{cos}}(\delta ){\text{sin}}(\omega_{{\text{s}}} )]$$

6 $${\text{d}}_{{\text{r}}} = {1 }0.0{33}\;{\text{cos}}\left( {\frac{2\pi }{{365}}{\text{J}}} \right)$$

7 $$\delta = 0.{4}0{\text{9 sin}}\left( {\frac{2\pi }{{365}}{\text{J}} - 1.39} \right)$$

8 $$\omega_{{\text{s}}} = {\text{arccos }}[ - {\text{tan }}(\varphi ){\text{ tan }}(\delta )]$$

9 $${\text{Radians}} = \pi /{18}0 \, \left( {\text{decimal degrees}} \right)$$

10 $${\text{R}}_{{{\text{nl}}}} = \sigma \left[ {\frac{{{\text{T}}_{{{\text{max}}}} {\text{k}}^{4} + {\text{T}}_{{{\text{min}}}} {\text{K}}^{4} }}{2}} \right]\left( {0.{34}{-}0.{14}\surd {\text{e}}_{{\text{a}}} } \right)\left[ {{1}.{35}\frac{{{\text{R}}_{{\text{s}}} }}{{{\text{R}}_{{{\text{so}}}} }} - 0.{35}} \right]$$

11 $${\text{R}}_{{{\text{so}}}} = (0.{75} + {2} \times { }10^{ - 5} {\text{Z}}){\text{ R}}_{{\text{a}}}$$

U_2_ is calculated from the following Eq. (12) recommended by Allen et al.^1^,

12 $${\text{U}}_{{2}} = {\text{U}}_{{\text{z}}} \frac{4.87}{{{\text{In}}\left( {67.8{\text{z}} - 5.42} \right)}}$$

where, R_ns_ is the net solar or shortwave radiation (MJ m^−2^ day^−1^), R_nl_ is the net outgoing longwave radiation (MJ m^−2^ day^−1^), R_s_ is the global solar or shortwave radiation (MJ m^−2 ^day^−1^), N and n are, respectively, the maximum and actual possible sunshine duration, R_a_ is the extraterrestrial radiation (MJ m^−2^ d^−1^), Gsc is the solar constant (0.0820 MJ m^−2^ min^−1^), d_r_ is the inverse relative distance Earth-Sun, ω_s_ is the sunset hour angle (rad), φ is latitude (rad), δ is solar declination (rad), J is the number of the day in the year between 1 (1 January) and 365 or 366 (31 December), σ is the Stefan-Boltzmann constant (4.903 × 10^−9^ MJ K^−4^ m^−2^ day^−1^), α is albedo (α = 0.23), T_max_k and T_min_k are, respectively, the maximum and minimum absolute temperatures during 24-h, and R_so_ is the clear sky solar radiation (MJ m^−2^ day^−1^). Allen et al.^1^ recommended 0.25 for a_s_ and 0.50 for b_s_. U_z_ is measured wind speed at Z_m_ above ground surface (ms^−2^) and z is respective station elevation above sea level (m).

According to Allen et al.^1^, Saturation Vapour Pressure (e_s_), Actual Vapour Pressure (e_a_), Slope Vapour Pressure Curve (∆) and Psychrometric Constant (γ) are calculated by the following Eqs. (13–19), respectively:

13 $${e}_{s}=\frac{{e}^{0}\left({T}_{max}\right) +{e}^{0}({T}_{min})}{2}$$

14 $${e}^{0}\left({T}_{max}\right)=0.6108\;exp\;\left[\frac{17.27{T}_{max}}{{T}_{max}+237.3}\right]$$

15 $${e}^{0}\left({T}_{min}\right)=0.6108\;exp\;\left[\frac{17.27{T}_{min}}{{T}_{min}+237.3}\right]$$

16 $${e}_{a}=\frac{Hr(mean)}{100}\left[\frac{{e}^{0}\left({T}_{max}\right) +{e}^{0}({T}_{min})}{2}\right]$$

17 $$\Delta =\frac{4098\left[0.6108 \;exp\;\left(\frac{17.27 T}{T+237.3}\right)\right]}{{\left(T+237.3\right)}^{2}}$$

18 $$\gamma = \frac{CpP}{{\varepsilon \lambda }} = 0.{665} \times {1}0^{{ - {3}}} {\text{P}}$$

19 $${\text{P}} = 101.3\left( {\frac{293 - 0.0065Z}{{293}}} \right)^{5.26}$$

where, es is the mean saturation vapour pressure (kPa), ${e}^{0}\left({T}_{max}\right) \mathrm{and}{ e}^{0}({T}_{min})$ are the saturation vapor pressure at maximum and minimum temperature, respectively. e_a_ is the actual vapour pressure function (kPa) and Hr is the mean relative humidity. T_ave_, T_max_ and T_min_ are the mean, maximum and minimum air temperature, respectively, in °C and exp [·] is 2.7183 (base of natural logarithm) raised to the power [··]. P is the atmospheric pressure (kPa), λ is the latent heat of vaporization (2.45 MJ kg^−1^), C_p_ is the specific heat at constant pressure (1.013 × 10–3 MJ kg^−1^ °C^−1^), ε is the ratio molecular weight of water vapour/dry air (0.622).

### Empirical models

A primary survey of literature clearly showed that the 13 ET_o_ empirical model performed usually well in various sub-regions worldwide. Abtew^51^, Jensen and Haise^52^, Irmak^53^, Makkink^54^, Priestley-Taylor^55^, Hargreaves-Samani^13^, Berti^56^, WMO^30^, Tabari^40^, and Turc^57^ models were chosen to compare to the FAO56-PM model. The 13 empirical models were chosen based on the available input meteorological variables, universal acceptance and their applicability worldwide (Table S1). The Hargreaves–Samani (HS), Hargreaves M1 (HM1), Hargreaves M2 (HM2) and Berti models used in this study, as the HS, HM1 and HM2 models require only the temperature and extraterrestrial radiation datasets and Berti model requires only temperature data, making these models less complex. Therefore, the 13 empirical models used in the present study can be classified into the three classes: four temperature-based model (Hargreaves–Samani, Hargreaves M1, Hargreaves M2, and Berti), one mass transfer-based models (WMO), eight radiation-based models (Makkink, Priestly–Taylor, Jensen–Haise, Abtew, Irmak1, Irmak2, Tabari, and Turc). The performances and application of these models had never been validated in Bangladesh so far. The studied models, input parameters, computed equations with references are outlined in Table 1.

**Table 1:** The original form of the 13 empirical models associated with the input parameters.

Sl. no.	Models	Models input	Equations	Proposed by
**Temperature-based**				
ET_o,1_	Hargreaves–Samani	R_a_, T_ave_, T_max_, T_min_	ET_0,1_ = [0.0023 × Ra (T_ave_ + 17.8) (T_max_ − T_min_)^0.5^]/λ	Hargreaves and Samani^13^
ET_o,2_	Hargreaves M1	R_a_, T_ave_, T_max_, T_min_	ET_0,2_ = [0.408 × 0.0030 × (T_ave_ + 20) (T_max_ − T_min_)^0.4^ × Ra	Hargreaves and Samani^13^
ET_o,3_	Hargreaves M2	R_a_, T_ave_, T_max_, T_min_	ET_o,3_ = $0.408\times 0.0023\times \left(Tave+17.8\right)\times {({T}_{max}-{T}_{min})}^{0.424}\times {R}_{a}$	Hargreaves and Samani^13^
ET_o,4_	Berti	R_a_, T_ave_, T_max_, T_min_	ET_0,3_ = $\left[0.00193 Ra\left({T}_{ave}+17.8\right){\left({T}_{max}-{T}_{min}\right)}^{0.517}\right]$/λ	Bertiet al.^56^
**Mass transfer-based**				
ET_o,5_	WMO	U_2_, e_s_ − e_a_	ET_0,4_ = (0.1298 + 0.0934U_2_)(e_s_ − e_a_)	WMO^30^
**Radiation-based**				
ET_o,6_	Abtew	R_s_, T_max_	ET_0,5_ = $\frac{1}{56}\frac{{R}_{s}{T}_{max}}{\lambda }$	Abtew^51^
ET_o,7_	Irmak1	R_s_, Tave	ET_0,6_ = $0.149{R}_{s}+0.079Tave-0.611$	Irmak et al.^53^
ET_o,8_	Irmak2	R_n_, T_ave_	ET_o,7_ = 0.489 + 0.289 R_n_ + 0.023 T_ave_	Irmak et al.^53^
ET_o,9_	Makkink	R_s_, T_ave_	ET0,8 = $0.61\frac{1}{\uplambda }\left[\frac{\Delta }{\Delta +\upgamma }\right]{\mathrm{R}}_{\mathrm{s}}-0.12$	Makkink^54^
ET_o,10_	Priestley-Taylor	R_n_, T_ave_	ET_0,9_ = $1.26\left[\frac{\Delta }{\Delta +\upgamma }\right]\left({\mathrm{R}}_{\mathrm{n}}-\mathrm{G}\right)/\uplambda$	Priestley and Taylor^55^
ET_o,11_	Jensen–Haise	R_s_, T_ave_	ET_0,10_ = $(0.025\mathrm{Tave}+0.08)\frac{{\mathrm{R}}_{\mathrm{s}}}{\uplambda }$	Jensen and Haise^52^
ET_o,12_	Tabari	R_s_, T_min_, T_max_	ET_0,11_ = 0.156R_s_—0.0112T_max_ + 0.0733T_min_—0.478	Tabari et al.^40^
ET_o,13_	Turc	R_s_, T_ave_	ET_o,13_ = 0.013 $\frac{{T}_{ave}}{{T}_{ave}+15}(Rs+50)$	Turc^57^

T_ave_, T_max_, and T_min_ are the mean (average), maximum, and minimum temperature (°C), respectively and λ is the latent heat of vaporization (2.45 MJ kg^−1^), ${\mathrm{a}}_{\mathrm{w}}=0.3+0.58\;{\mathrm{exp}}\;\left[-{\left(\frac{\mathrm{J}-170}{45}\right)}^{2}\right]$ and ${\mathrm{b}}_{\mathrm{w}}=0.32+0.54\;{\mathrm{exp}}\;\left[-{\left(\frac{\mathrm{J}-228}{67}\right)}^{2}\right]$ (after Peng et al.^11^).

### Performance evaluation of 13 empirical models

Performance evaluation of 13 empirical models, based on the accuracy of each model for estimating ET_o_, was undertaken by six statistical criteria. The six statistical criteria were the mean bias error (MBE)^58^; mean absolute error (MAE), correlation of determination (R^2^), root mean square error (RMSE)^43^; relative error (RE), Nash–Sutcliffe efficacy coefficient (NSE)^59^ expressed by the following Eqs. (20–25):

20 $$MAE=\frac{1}{n}\sum_{i=1}^{n}\left|{ET}_{o,s}-{ET}_{o,i}\right|$$

21 $$MBE=\frac{1}{n}\sum_{i=1}^{n}{ET}_{o,i}-{ET}_{o,s}$$

22 $$NSE=1-\frac{\sum_{i=1}^{n}{({ET}_{o,s}-{ET}_{o,i})}^{2}}{\sum_{i=1}^{n}{({ET}_{o,s}-\stackrel{-}{{ET}_{o,s})}}^{2}}$$

23 $${R}^{2}=\frac{\sum_{i=1}^{n}{({ET}_{o,s}-{ET}_{o,i})}^{2}}{\sum_{i=1}^{n}{({ET}_{o,s}-{\stackrel{-}{ET}}_{o,i})}^{2}}$$

24 $$RE=\frac{{ET}_{o,i}-{ET}_{o,s}}{{ET}_{o,s}}$$

25 $$RMSE=\sqrt{\frac{1}{n}\sum_{i=1}^{n}{({ET}_{o,s}-{ET}_{o,i})}^{2}}$$

where, ${\mathrm{ET}}_{\mathrm{o},\mathrm{s}}$, ${\mathrm{ET}}_{\mathrm{o},\mathrm{i}}$ and n are the observed ET_o_ (estimated by FAO56-PM), estimated ET_o_ (estimated by empirical models) and total observations, respectively.

### Modified Mann–Kendall test

Modified Mann–Kendall (MMK) test is a non-parametric test which was applied for detecting the increasing and decreasing trend of ET_o,s_ in Bangladesh during 1980–2017^60^. To carry out this MKK test, it is imperative to confirm the serial autocorrelation of the time series dataset. Hence, the serial autocorrelation should be excluded before employing the MMK test. To exclude the serial autocorrelation, the trend free pre-whitening approach proposed by Yue and Wang^61^ has been utilized. The original form of MK test^62,63^ statistics (S) is as followed:

26 $$S=\sum_{i=1}^{n-1}\sum_{j=i+1}^{n}sgn({X}_{j}-{X}_{i})$$

27 $$sgn\left( \theta \right) = \left\{ {\begin{array}{*{20}l} 1 \hfill & {if\;\theta > 0} \hfill \\ 0 \hfill & {if\;\theta = 0} \hfill \\ { - 1} \hfill & {if\;\theta = 0} \hfill \\ \end{array} } \right.$$

Direction of increasing or decreasing trend is indicated by S. The variance^64^ of S is followed by the Eq. (29):

28 $$V\left(S\right)=\frac{n\left(n-1\right)\left(2n+5\right)-\sum_{i=1}^{n}{t}_{i}i\left(i-1\right)(2{t}_{i}+5)}{18}$$

V*(S), is the modified variance^61^ given by following Eq. (30):

29 $${V}^{*}\left(S\right)=V\left(S\right)\cdot \frac{n}{{n}^{*}}$$

n/n* is termed as correction factor and denoted^65^ by following Eq. (31):

30 $$\frac{n}{{n^{*} }} = 1 + 2 \cdot \mathop \sum \limits_{k = 1}^{n - 1} \left( {1 - \frac{k}{n}} \right) \cdot \rho_{k}$$

Test statistic Z is calculated by following Eq. (32):

31 $$Z = \left\{ {\begin{array}{*{20}l} {\frac{{S - 1}}{{\sqrt {V^{*} (S)} }}} &\quad {S > 0} \\ 0 &\quad {S = 0} \\ {\frac{{S + 1}}{{\sqrt {V^{*} (S)} }}} &\quad {S < 0} \\ \end{array} } \right.$$

Positive Z statistic indicates increasing trend of ET_o,s_ and negative Z statistic indicates decreasing trend of ET_o,s_ in Bangladesh.

### Sen’s slope of estimator

Sen’s slope of estimator^66^ was applied for calculating the change of ET_o,s_ in Bangladesh per decade and the statistics is as followed:

32 $${\text{Q}} = xj-xkj$$

Q is denoted as the slope between *xj* and *xk*.

The spatial distributions of the monthly *ET*_o_ and its trends are mapped.

Spatial distributions of the monthly meteorological variables; ET_o,s_, ET_o,i_, trends and the other examined variables are mapped by the inverse distance weighted interpolation model in ArcGIS 10.5 software.

### Random forest (RF) model

The RF is a heuristic decision tree-based supervised machine learning model^67^ that is appropriate for addressing the existence of the over-fitting problem to the decision trees, and other machine learning algorithm^68^. The RF is most robust, can handle numerous heterogeneous covariates, and has been effectively employed into the hydrological field^69^, genetic engineering field^70^ and hydro-meteorological field^71^. The RF model has been benefited from the two more powerful algorithms e.g., bagging and random binary trees, which are called the powerhouse of this model. For developing the RF model, the number of trees and features in each split is essential. RF is a classifier which comprises of an assortment of classifier trees ${f}_{m}(x)$ for m = 1, …, M which relies on the parameters and every single tree casts a unit vote for input x^71^. Each tree generates an individual class which then combined and the majority vote predicts the final results. Present study optimized its accuracy with 100 trees, 1 execution slot, 5 seeds and with maximum depth 1. As a tree-based ensemble learning model, this model has extensively used to evaluate the importance degree of any climatic dataset in various regions^25,72^. To the best of author’s knowledge, the RF model has not yet been employed to explore the importance degree of 13 empirical models against the FAO56-PM model in Bangladesh. The RF model is used to know which model is most reliable and dominant for estimating ET_o_. More detailed about the RF model can be found elsewhere^46,71^.

## Results

### Spatial distribution of meteorological variables

**Figure 2:**
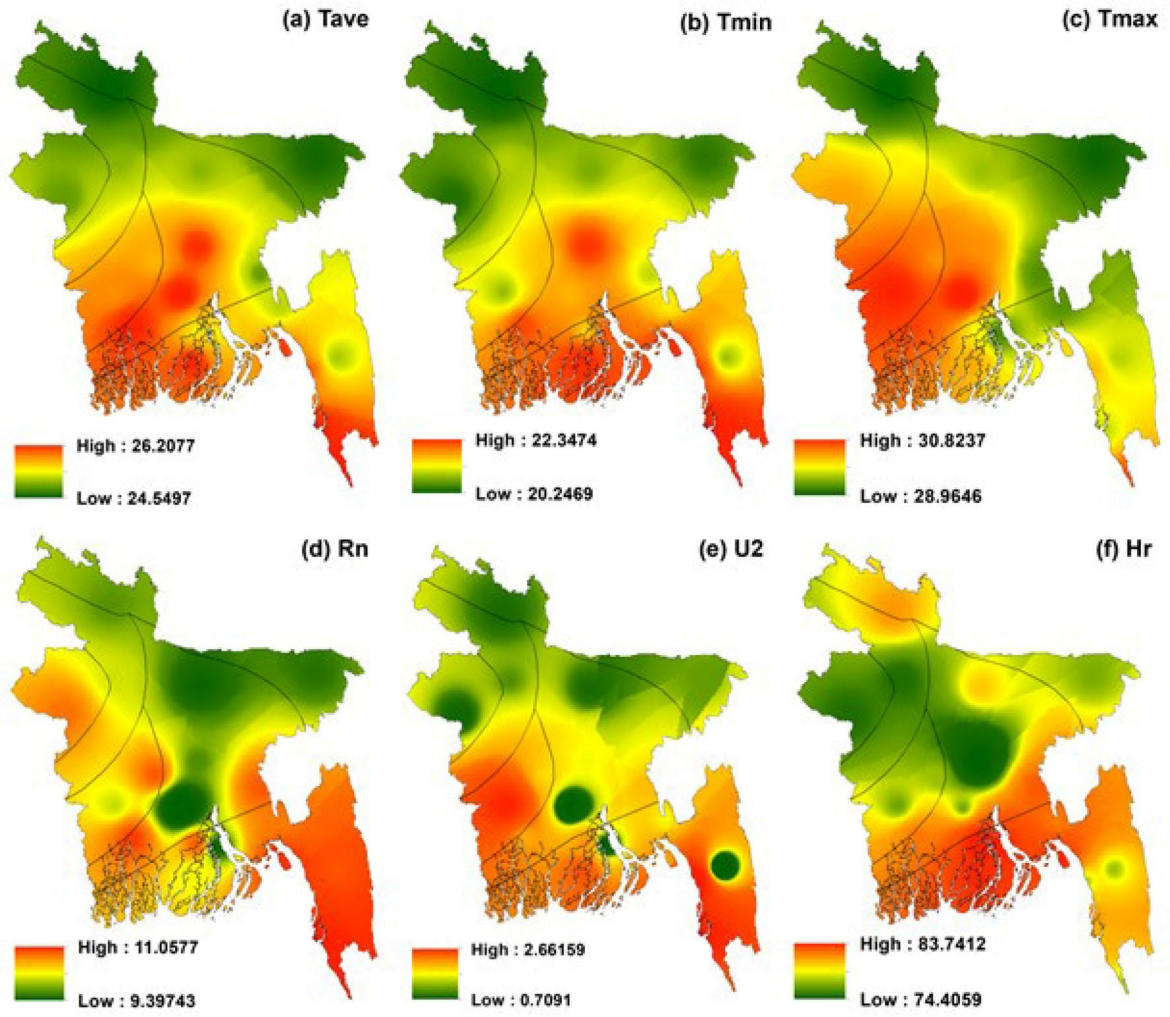
Spatial distribution of multi-year mean meteorological variables in Bangladesh, prepared by ArcGis 10.5 (www.esri.com).

Figure 2 represents the distribution of multi-year mean meteorological variables of T_ave_, T_min_, T_max_, Rn, U2, and Hr from 1980 to 2017. Distribution of T_ave_ (Fig. 2a), T_min_ (Fig. 2b) and T_max_ (Fig. 2c) showed almost similar results. Sub-region VI showed the higher values of historical temperature, while sub-regions II and III entirely showed the lowest temperature. Sub-region V showed the lowest temperature for the distribution of T_ave_ and T_min_ and moderate temperature for T_max_. Sub-regions I, IV and VII showed the moderate values. The higher rate of net radiation was observed in the sub-region I and lower rate of net radiation was found in the sub-regions II and III (Fig. 2d). Average values of Rn were seen in the sub-regions IV, V, VI, and VII. The highest wind speed was found in the sub-region of VI and the lowest in the sub-regions II, III and V (Fig. 2e). Sub-region I experienced a comparatively higher rate of Hr as it located near the Bay of Bengal from where this region took available moisture and sub-region V experienced the lower rate of Hr (Fig. 2f).

### Spatial and temporal patterns of ET_o,s_ and ET_o,i_

**Figure 3:**
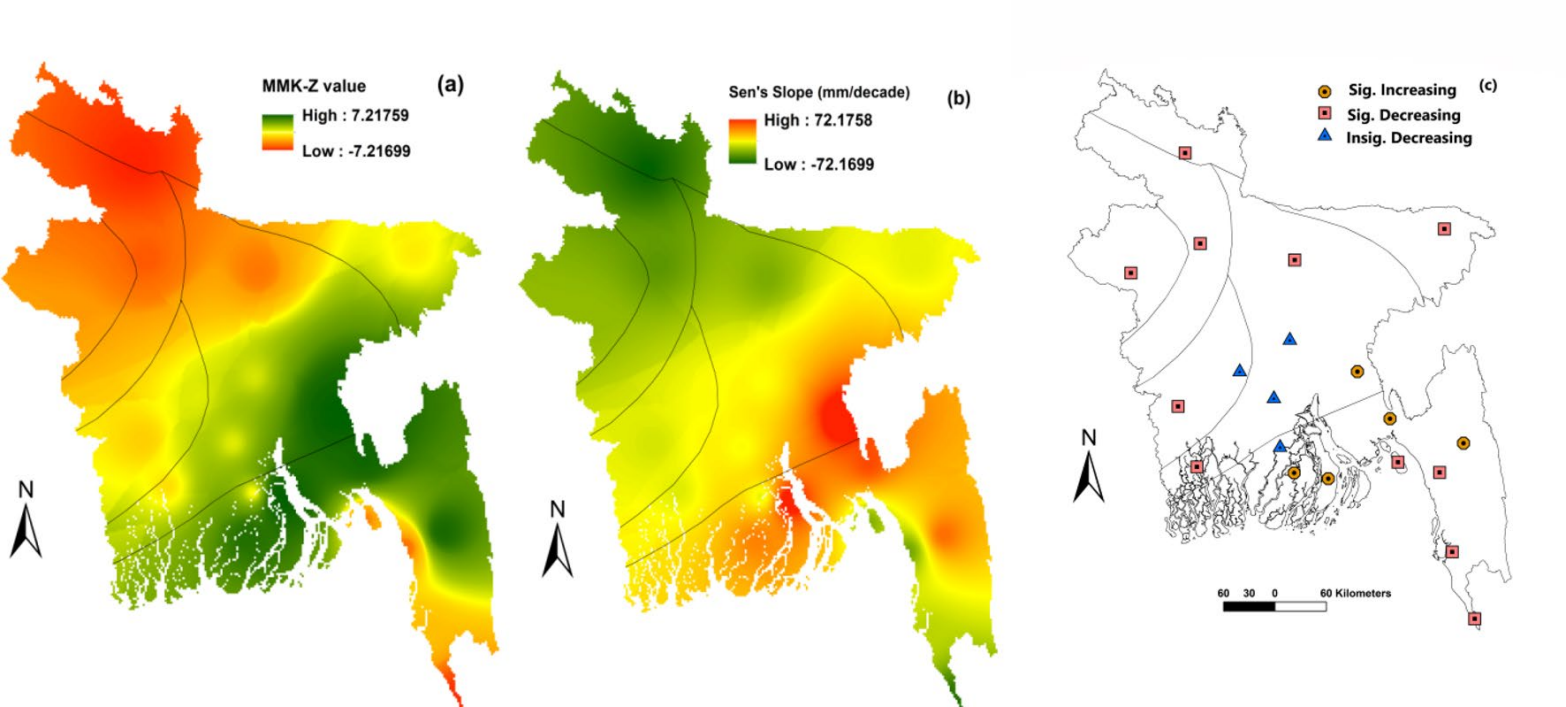
Representation of the multi-year mean monthly ET_o,s_ trends of (**a**) MMK-Z values (mm); (**b**) Sen’s slope estimation and (**c**) station wise increasing or decreasing trends of Bangladesh, prepared by ArcGis 10.5 (www.esri.com).

Figure 3 represents the long-term multi-year mean monthly trends of ET_o,s_ (FAO56-PM). Most parts of sub-regions I and VII showed the higher values of MMK-Z statistic and the sub-regions III, IV and V showed the lowest values of MMK-Z statistic (Fig. 3a). Moderate values are shown in the sub-regions II and VI. In general, the rates between increasing and decreasing of ET_o,s_ was from 72.18 to − 72.17 mm per decade (Fig. 3b). Figure 3c shows the nature of the trend whether it was significant or insignificant. The significant increasing trend of ET_o,s_ was detected in Bhola, Cumilla, Feni ($\alpha = 0.01$); Rangamati, and Patuakhali ($\alpha = 0.05$) in the sub-regions I and VII.

All the weather stations of sub-regions II ($\alpha = 0.1$); III, IV ($\alpha = 0.01$) and V ($\alpha = 0.05$) showed a significant decreasing trend of ET_o,s_. Faridpur, Madaripur, Dhaka and Barishal of sub-regions I and VII showed an insignificant decreasing trend of ET_o,s_. Cox’s Bazar ($\alpha = 0.05$); Teknaf, Sandwip and Chattogram ($\alpha = 0.01$) of sub-region I; Jashore ($\alpha = 0.05$) of sub-region VI and Mymensingh ($\alpha = 0.01$) and Khulna ($\alpha = 0.1$) of sub-region VII showed a significant decreasing trend of ET_o,s_.

**Figure 4:**
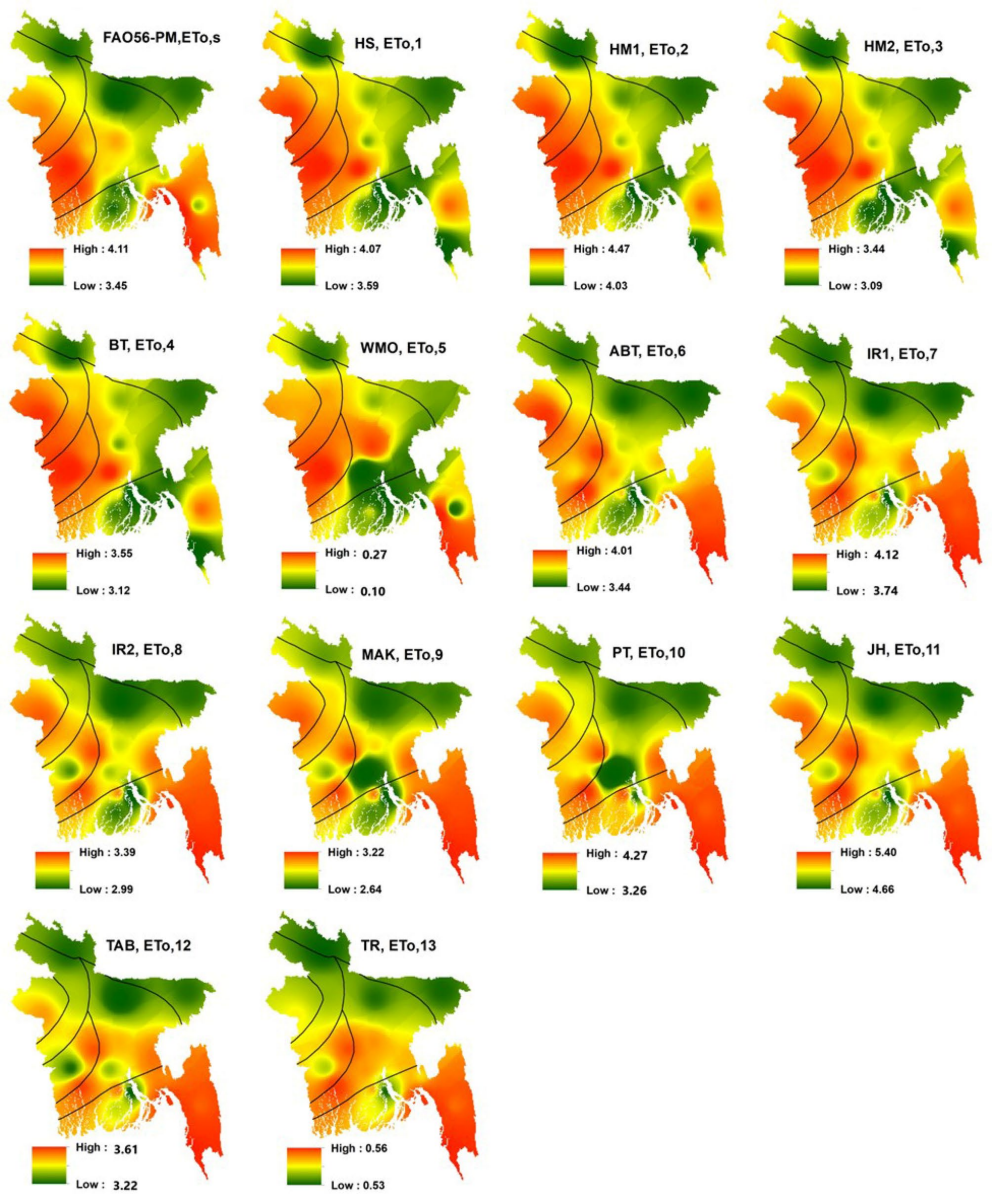
Spatial distribution of multi-year mean monthly ET_o,s_ and ET_o,i_ in Bangladesh, prepared by ArcGis 10.5 (www.esri.com).

Spatial distribution of multi-year mean monthly ET_o,s_ and ET_o,i_ from 1980 to 2017 is presented in Fig. 4. The higher value (4.12 mm) of ET_o,s_ seen in the sub-regions V, VI and some parts of region I. The lower value (3.46 mm) of ET_o,s_ seen in the sub-regions II, III and some part of the sub-regions I, IV and VII. The distribution of ET_o,i_ showed the homogeneous distribution of ET_o,s_. The high-low values of spatial distribution of ET_o,1_ (temperature-based); ET_o,6_; ET_o,7_; ET_o,10_ (radiation-based) models were analogous with that of ET_o,s_. ET_o,5_ (mass transfer-based) and ET_o,13_ (radiation-based) models showed the most heterogeneity with ET_o,s_. Sub-regions V and VI showed the highest value of ETo (computed by most of the empirical models and FAO56-PM) and all the models found that the sub-regions II and III experienced a lower rate of ETo. All the models revealed that moderate ETo was experienced by the sub-regions I, IV and VII. Approximately, similar zonation (based on the highest and lowest values) was observed between ET_o,s_ and ET_o,6_.

**Figure 5:**
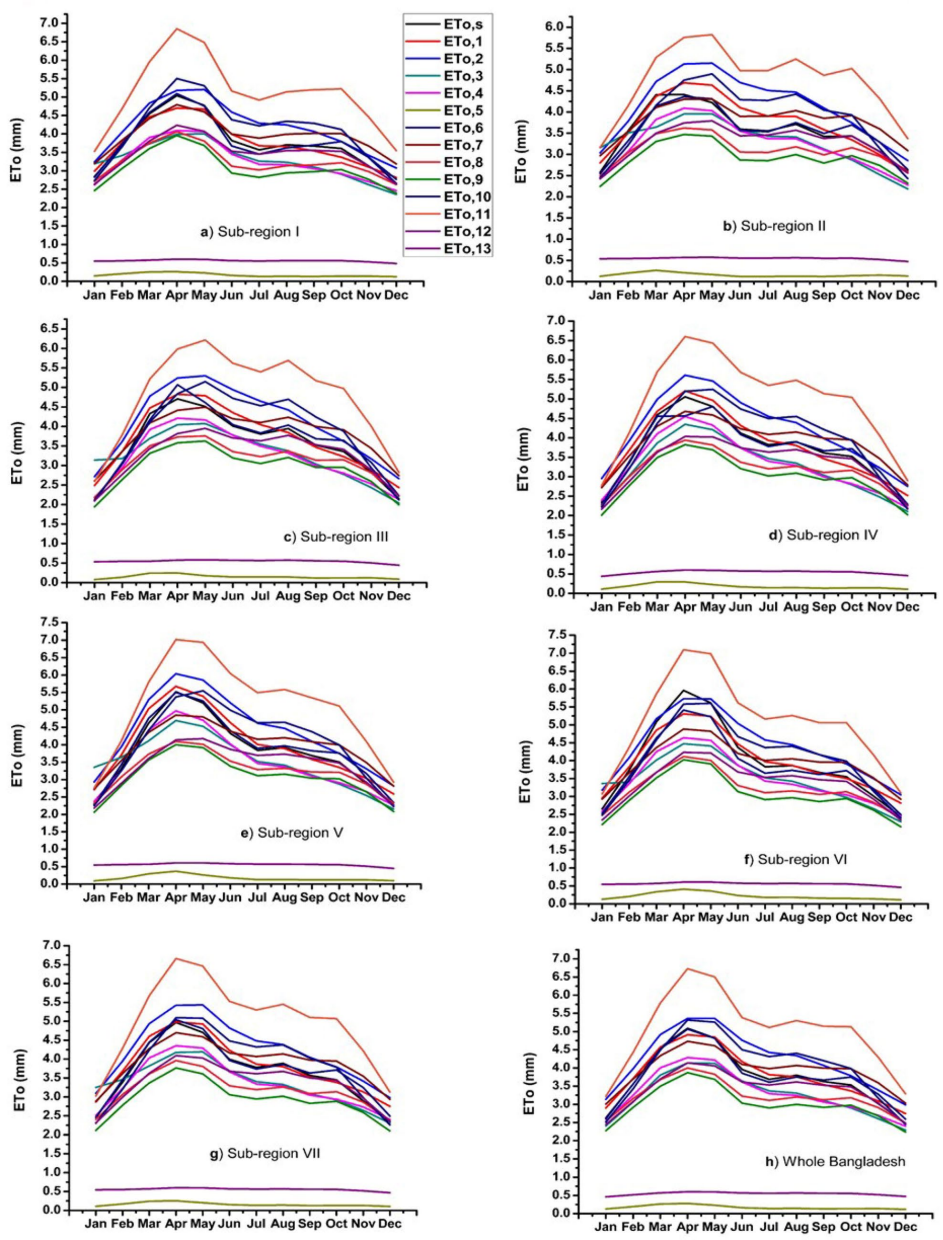
Temporal distribution of multi-year mean monthly ET_o,s_ and ET_o,i_ in different sub-regions and whole Bangladesh.

Temporal distribution of long-term monthly ET_o,s_, and ET_o,i_ in different sub-regions, as well as whole Bangladesh for the period of 1980–2017, is shown in Fig. 5. The highest rate of reference evapotranspiration (ET_o,s_ and ET_o,i_) occurred in April in all the regions of Bangladesh. The lowest ETo (both ET_o,s_ and ET_o,i_) found in January and December. Except for sub-regions II and III, the range of the rate of ET_o,s_, and ET_o,i_ was the same in all the sub-regions. Among the 13 empirical models, ET_o,5_; ETo13 (elevated the lowest values than ET_o,s_) and ET_o,11_ (elevated the highest values than ET_o,s_) models showed the most deficit values of ET_o,i_ compared to ET_o,s_ identifying least suitable method for estimating ETo. Conversely, the values estimated by ET_o,6_ showed the closest values to ET_o,s_ demonstrating as the most preferable model for estimating ETo. ET_o,1_ and ET_o,7_ also estimated values with the smallest difference with ET_o,s_ in all the regions, confirmed as the preferable method for estimating ETo. Moderate values estimated by ET_o,2_; ET_o,4_; ET_o,8_; ET_o,9_; ET_o,10_ and ET_o,12_ models compared to ET_o,s_.

**Figure 6:**
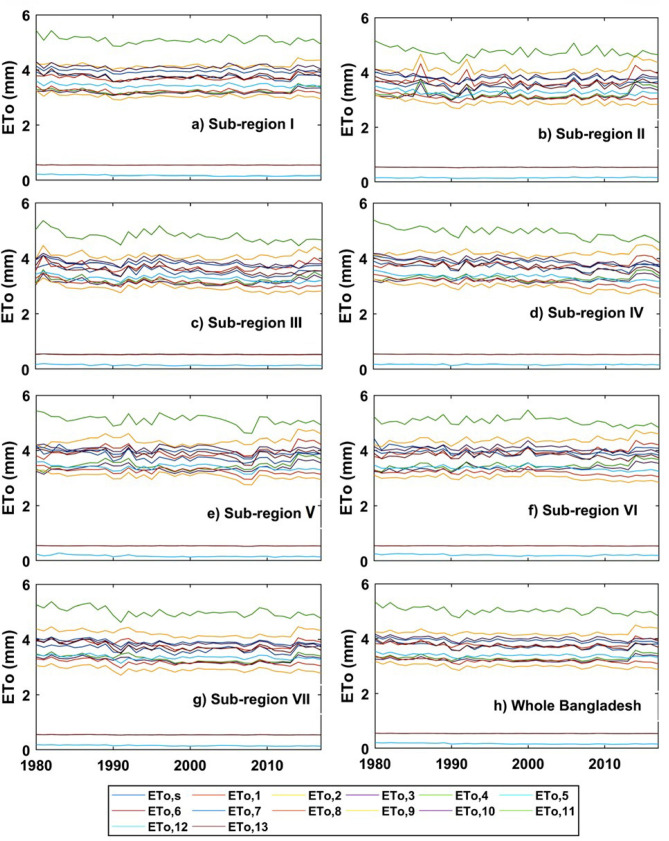
The inter-annual variations of ET_o,s_ and ET_o,i_ in different sub-regions and whole Bangladesh.

Long term inter-annual variation of ET_o,s_, and ET_o,i_ from 1980 to 2017 are represented by Fig. 6. It also demonstrates similar results as shown in Fig. 5. The largest deviation from the ET_o,s_ values occurred by estimating ET_o,i_ (values lower than 1) by both ET_o,5_ and ET_o,13_ models. The Fig. 6 shows that the rate of ETo in each sub-region along with whole Bangladesh, estimated by both ET_o,s_ and ET_o,i_ models, declined gradually. Unlike the multi-year monthly distribution, the range (from 0 to 5.5 mm) of ETo values was nearly similar in every sub-regions and Bangladesh. Like the multi-year monthly distribution, ET_o,6_; ET_o,1_; ET_o,7_ and ET_o,10_ showed the very closest values to ET_o,s_ in each sub-region. Values larger than that of ET_o,s_ found by ET_o,11_. Based on the spatiotemporal distribution of ET_o,i_ estimated by 13 empirical models in each sub-region and whole Bangladesh, the empirical models can be ranked in ascending order based on the closest values to ET_o,s_ as ET_o,6_ > ET_o,1_ > ET_o,7_ > ET_o,10_ > ET_o,3_ > ET_o,2_ > ET_o,12_ > ET_o,4_ > ET_o,8_ > ET_o,9_ > ET_o,11_ > ET_o,13_ > ET_o,5_.

### Performance appraisal of 13 empirical models for estimating ETo

**Figure 7:**
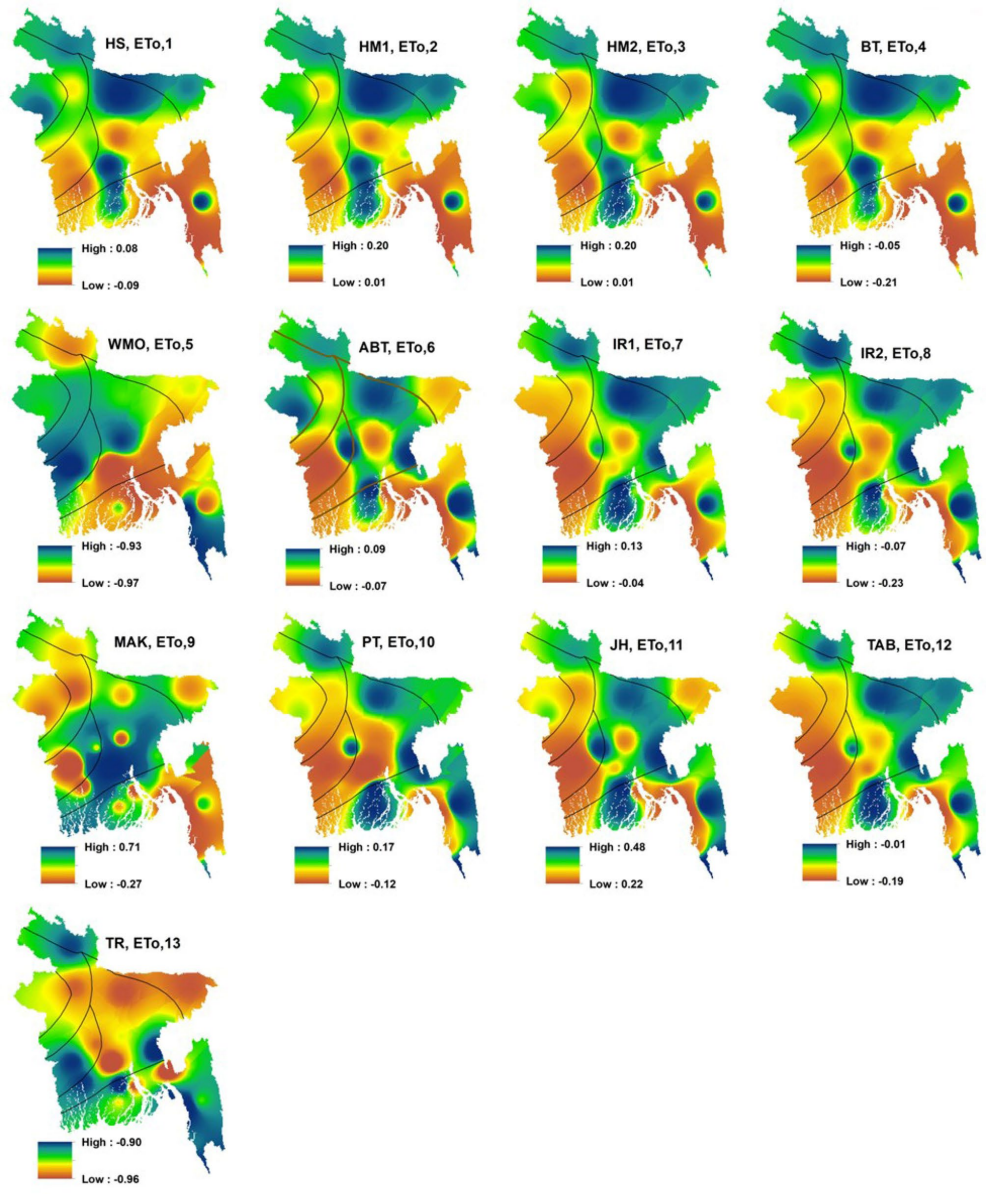
Spatial distribution of relative error values for multi-year mean monthly ET_o,i_ in Bangladesh, prepared by ArcGis 10.5 (www.esri.com).

Figure 7 shows the long term monthly RE of ET_o,i_ by spatial distribution for the period of 1980–2017. RE of the maximum ET_o,i_ covered mutually positive and negative values. The lowest RE was observed in ET_o,i_ calculated by ET_o,6_. Furthermore, ET_o,1_; ET_o,7_; ET_o,10_ and ET_o,12_ models also produced lower RE, respectively. The worst performance with the high RE belongs to the ET_o,5_ and ET_o,13_ models. The relative error in different sub-regions varied with the variation of different empirical models. The ET_o,11_, ET_o,9_, ET_o,8_, ET_o,2_, ET_o,3_ and ET_o,4_ models also recognized as the worst models, respectively, producing high RE.

Table 2 shows the long term mean monthly and annual RMSE for 13 selected ET_o,i_ models for whole Bangladesh. The higher RMSE was produced by the ET_o,5_; ET_o,13_ and ET_o,11_ models, respectively. The ET_o,6_, ET_o,3_, ET_o,7_ and ET_o,1_ models generated the least RMSE for calculating ETo. The descending order of the other models based on performance was ET_o,2_ > ET_o,9_ > ET_o,4_ > ET_o,10_ > ET_o,8_ > ET_o,12_.

**Table 2:** Root Mean Square Error (RMSE) values of the 13 empirical models for calculating ET_o,i_ at the monthly and annual timescales for Bangladesh. The bold values indicate the optimal model among other models.

ET_o,i_	ET_0,1_	ET_0,2_	ET_0,3_	ET_0,4_	ET_0,5_	ET_0,6_	ET_0,7_	ET_0,8_	ET_0,9_	ET_0,10_	ET_0,11_	ET_0,12_	ET_0,13_
**Month/year**													
Jan	0.8428	0.6065	0.2472	1.2001	3.6024	**0.6445**	0.7391	1.2053	1.4635	1.2277	0.5630	1.2919	2.1696
Feb	0.1778	0.2978	0.5039	0.5073	3.5384	**0.1224**	0.1562	0.5903	0.8282	0.3469	0.7474	0.6696	3.0446
Mar	0.8772	1.2064	0.7722	0.3474	3.4644	**0.5213**	0.6215	0.1384	0.2671	0.7748	2.0732	0.1221	3.9983
Apr	1.2023	1.6439	0.9778	0.5885	3.4517	**0.1097**	1.0133	0.3032	0.2066	1.5978	3.0151	0.4335	4.4995
May	1.1439	1.6483	0.7211	0.5334	3.4982	**0.2788**	0.9108	0.2805	0.2841	1.5718	2.8234	0.3941	4.2382
Jun	0.5031	1.0590	0.3541	0.2434	3.5659	**0.2386**	0.4159	0.5439	0.7337	0.8229	1.7057	0.3194	3.3993
Jul	0.3903	0.7151	0.3648	0.4633	3.5911	**0.2045**	0.3112	0.6522	0.8568	0.6409	1.4344	0.2689	3.1328
Aug	0.3050	0.6421	0.5209	0.5180	3.5873	**0.2181**	0.3912	0.5703	0.7650	0.7311	1.6220	0.2223	3.2419
Sep	0.2573	0.3774	0.5441	0.6840	3.5962	**0.2237**	0.2989	0.6237	0.8279	0.5066	1.4465	0.2449	3.0685
Oct	0.4074	0.1889	0.6452	0.8218	3.5926	**0.2847**	0.3048	0.5850	0.7819	0.3394	1.4457	0.2989	2.9804
Nov	0.6533	0.3651	0.4289	1.0397	3.5916	**0.1981**	0.2054	0.8368	1.0631	0.5793	0.6273	0.7131	2.5004
Dec	0.9907	0.7483	0.2197	1.3340	3.6142	**0.1554**	0.7190	1.2593	1.5035	1.2897	0.5162	1.2602	2.0195
Year	0.1045	0.4872	0.5062	0.4441	3.5580	**0.0484**	0.2064	0.5317	0.7337	0.2805	1.3012	0.3444	3.1848

Table 3 represents the long term mean monthly and annual MAE values of 13 empirical models used for estimating ET_o,i_. Lower values of MAE indicates higher accuracy. Respectively, ET_o,6_ and ET_o,1_ models produced the smallest MAE values for both the monthly and annual ET_o,i_ values in Bangladesh. Higher MAE values produced by the models (respectively) of ET_o,5_, ET_o,13_, ET_o,11_, ET_o,9_ and ET_o,8_ in calculating both the monthly and annual ET_o,i_.

**Table 3:** Mean Absolute Error (MAE) values of 13 empirical models for calculating ET_o,i_ at the monthly and annual timescales for Bangladesh. The bold values indicate the optimal model among other models.

ET_o,i_	ET_0,1_	ET_0,2_	ET_0,3_	ET_0,4_	ET_0,5_	ET_0,6_	ET_0,7_	ET_0,8_	ET_0,9_	ET_0,10_	ET_0,11_	ET_0,12_	ET_0,13_
**Month/year**													
Jan	0.398	0.562	0.222	0.273	2.497	**0.260**	0.425	0.251	0.384	0.256	0.613	0.264	2.163
Feb	0.369	0.716	0.489	0.387	3.363	**0.293**	0.306	0.416	0.643	0.309	0.879	0.491	3.039
Mar	0.361	0.471	0.760	0.601	4.296	**0.320**	0.336	0.884	1.063	0.389	1.232	0.866	3.992
Apr	0.443	0.470	0.952	0.831	4.814	**0.330**	0.411	1.095	1.222	0.488	1.640	0.954	4.491
May	0.374	0.605	0.694	0.646	4.591	**0.315**	0.314	1.000	1.137	0.575	1.681	0.770	4.227
Jun	0.350	0.810	0.318	0.415	3.794	**0.231**	0.244	0.736	0.930	0.593	1.428	0.366	3.391
Jul	0.305	0.742	0.320	0.457	3.546	**0.187**	0.309	0.578	0.786	0.651	1.430	0.200	3.125
Aug	0.285	0.557	0.493	0.575	3.657	**0.190**	0.297	0.600	0.800	0.644	1.503	0.220	3.235
Sep	0.287	0.473	0.521	0.581	3.490	**0.174**	0.381	0.503	0.707	0.599	1.524	0.164	3.063
Oct	0.283	0.340	0.631	0.615	3.394	**0.296**	0.468	0.359	0.555	0.505	1.600	0.167	2.974
Nov	0.280	0.436	0.413	0.365	2.880	**0.336**	0.584	0.213	0.356	0.288	1.281	0.204	2.496
Dec	0.366	0.538	0.196	0.244	2.370	**0.262**	0.565	0.232	0.296	0.232	0.819	0.221	2.013
Year	0.226	0.500	0.500	0.448	3.557	**0.178**	0.244	0.528	0.731	0.343	1.299	0.341	3.183

Table 4 shows the NSE coefficient of empirical models used for calculating ET_o,i_ at the annual and monthly time scale in Bangladesh. Approximately, all the models gave negative NSE value indicating the least correlated method with ET_o,s_. In both monthly and annual timescales, ET_o,6_ outperformed as it gave positive NSE value, except for the month of October and November. The performance accuracy of ET_o,1_ was comparatively higher than that of other models except for ET_o,6_, ET_o,13_, ET_o,5_ and ET_o,11_ models. In annual timescale, the order of the performance of the models was ET_o,6_ > ET_o,7_ > ET_o,1_ > ET_o,10_ > ET_o,12_ > ET_o,4_ > ET_o,2_ > ET_o,8_ > ET_o,9_ > ET_o,3_ > ET_o,11_ > ET_o,5_ > ET_o,13_.

**Table 4:** Nash–Sutcliffe efficiency (NSE) coefficients of the 13 empirical models for calculating ET_o,i_ at the monthly and annual timescales for Bangladesh. The bold values indicate the optimal model among other models.

ET_o,i_	ET_0,1_	ET_0,2_	ET_0,3_	ET_0,4_	ET_0,5_	ET_0,6_	ET_0,7_	ET_0,8_	ET_0,9_	ET_0,10_	ET_0,11_	ET_0,12_	ET_0,13_
**Month/year**													
Jan	− 0.274	− 1.258	− 1.033	0.123	− 35.578	**0.255**	− 0.39	0.354	− 0.302	0.336	− 1.958	0.258	− 155.576
Feb	− 0.103	− 2.353	− 5.205	− 0.426	− 58.766	**0.238**	0.312	− 0.449	− 1.77	0.166	− 3.821	− 0.828	− 225.502
Mar	− 0.093	− 0.559	− 9.619	− 1.523	− 89.31	**0.142**	0.003	− 3.595	− 5.338	− 0.23	− 7.404	− 3.525	− 283.656
Apr	− 0.065	− 0.089	− 10.441	− 1.993	− 74.489	**0.362**	− 0.061	− 3.579	− 4.554	− 0.105	− 8.519	− 2.672	− 241.254
May	0.187	− 0.754	− 4.042	− 1.022	− 72.584	**0.354**	0.338	− 3.021	− 4.07	− 0.452	− 9.741	− 1.603	− 173.139
Jun	− 0.127	− 3.507	− 1.077	− 0.462	− 81.971	**0.38**	0.467	− 2.519	− 4.436	− 1.483	− 11.67	− 0.159	− 190.445
Jul	− 0.495	− 5.005	− 1.591	− 1.531	− 109.474	**0.436**	− 0.093	− 2.268	− 4.824	− 3.206	− 17.811	0.422	− 190.097
Aug	− 0.233	− 2.778	− 4.285	− 2.871	− 121.219	**0.351**	− 0.03	− 2.678	− 5.35	− 3.301	− 20.738	0.263	− 203.639
Sep	− 0.415	− 2.248	− 7.579	− 3.418	− 124.944	**0.419**	− 0.781	− 1.972	− 4.612	− 3.229	− 23.887	0.48	− 271.754
Oct	− 0.481	− 0.762	− 9.322	− 3.902	− 119.488	− **0.355**	− 1.729	− 0.846	− 2.843	− 2.18	− 26.957	0.439	− 219.231
Nov	− 0.309	− 1.496	− 7.088	− 1.192	− 82.924	− **0.672**	− 2.958	0.103	− 0.97	− 0.236	− 16.758	0.266	− 273.802
Dec	− 0.371	− 1.721	− 0.683	0.106	− 42.958	**0.047**	− 1.95	0.255	− 0.226	0.281	− 5.249	0.311	− 141.118
Year	− 0.477	− 4.116	− 25.07	− 3.474	− 211.012	**0.176**	− 0.341	− 4.411	− 8.788	− 1.498	− 28.244	− 1.667	− 1030.942

**Figure 8:**
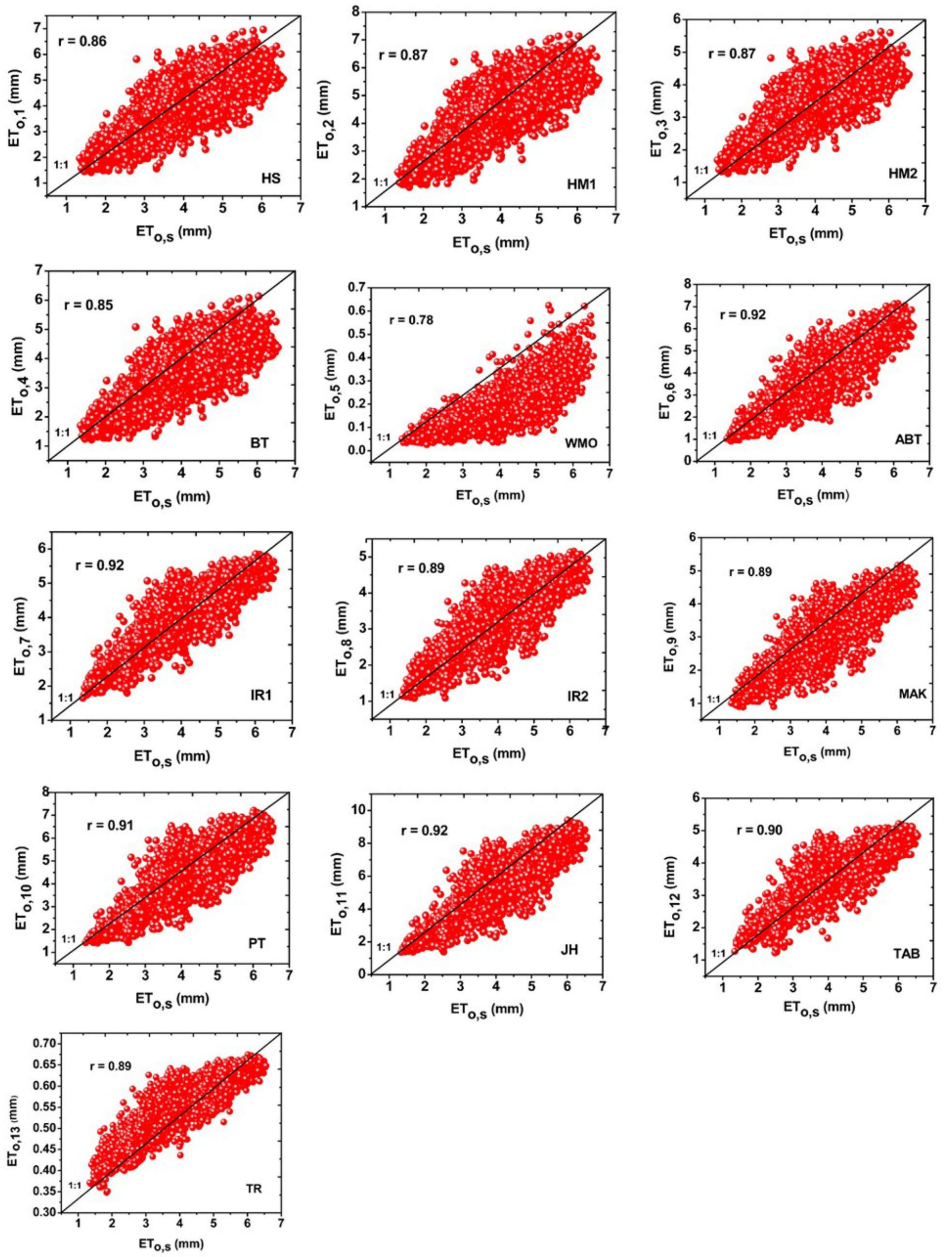
Scatter plots showing the comparisons of daily ET_o,i_ and ET_o,s_ in Bangladesh.

MBE at both long term annual and monthly time scale in calculating ET_o,i_ in Bangladesh is shown in Table 5. ET_o,6_ and ET_o,1_ showed higher performance accuracy, producing lower MBE values. Again, ET_o,5_; ET_o,13_ and ET_o,11_ models showed the least performance accuracy for calculating ET_o,i_. Other models showed moderate performance accuracy in estimating ET_o,i_. From the above discussions of performance accuracy, ET_o,6_ revealed as the suitable alternative model for estimating ETo in Bangladesh. Whereas, ET_o,5_; ET_o,13_ and ET_o,11_models explored as the worst performed empirical models inappropriate for estimating ET_o_ in Bangladesh. Figure 8 shows the scatter plots of daily ET_o,s_ vs. ET_o,i_ of Bangladesh during 1980–2017. The ET_o,6_; ET_o,7_ and ET_o,11_ models outperformed among 13 empirical models with higher r-value (0.92). ET_o,10_ (r-value 0.91) and ET_o,12_ (r-value 0.90) performed well, respectively, which produced r-values ≥ 0.90.

**Table 5:** Mean Bias Error (MBE) values of the 13 empirical models for calculating ET_o,i_ at the monthly and annual timescales for Bangladesh. The bold values indicate the optimal model among other models.

ET_o,i_	ET_0,1_	ET_0,2_	ET_0,3_	ET_0,4_	ET_0,5_	ET_0,6_	ET_0,7_	ET_0,8_	ET_0,9_	ET_0,10_	ET_0,11_	ET_0,12_	ET_0,13_
**Month/year**													
Jan	0.271	0.509	− 0.222	− 0.090	− 2.497	− **0.025**	0.380	− 0.091	− 352	− 0.118	0.591	− 0.181	− 2.163
Feb	0.153	0.654	− 0.489	− 0.307	− 3.363	**0.002**	0.138	− 0.398	− 0.640	− 0.129	0.871	− 0.482	− 3.039
Mar	0.011	0.354	0.787	− 0.561	− 4.296	− **0.043**	− 0.225	− 0.883	− 1.063	− 0.073	1.225	− 0.865	− 3.995
Apr	− 0.179	0.269	0.945	− 0.806	− 4.814	− **0.024**	− 0.360	− 1.094	− 1.222	0.224	1.636	− 0.952	− 4.498
May	0.025	0.540	0.690	− 0.603	− 4.591	− **0.009**	− 0.213	− 0.999	− 1.137	0.438	1.679	− 0.767	− 4.230
Jun	0.215	0.805	0.315	− 0.346	− 3.794	− **0.101**	0.137	− 0.731	− 0.927	0.542	1.427	− 0.343	− 3.397
Jul	0.129	0.740	0.311	− 0.392	− 3.546	− **0.08**	0.294	− 0.578	− 0.786	0.628	1.430	− 0.153	− 3.120
Aug	− 0.043	0.541	0.482	− 0.555	− 3.657	− **0.050**	0.268	− 0.600	− 0.800	0.604	1.503	− 0.187	− 3.230
Sep	− 0.081	0.442	0.531	− 0.559	− 3.490	**0.043**	0.370	− 0.500	− 0.707	0.574	1.524	− 0.100	− 3.055
Oct	− 0.161	0.254	0.625	− 0.605	− 3.394	**0.218**	0.444	− 0.351	− 0.554	0.454	1.600	− 0.051	− 2.956
Nov	0.074	0.371	0.421	− 0.320	− 2.880	**0.261**	0.572	− 0.109	− 0.338	0.154	1.280	0.013	− 2.356
Dec	0.258	0.502	0.187	− 0.087	− 2.370	**0.107**	0.546	− 0.002	− 0.249	− 0.038	0.804	− 0.006	− 2.113
Year	0.056	0.480	− 0.500	− 0.436	− 3.557	**0.025**	0.198	− 0.528	− 0.731	0.274	1.299	− 0.338	− 3.283

All the scatter plots produced r values with significant p-value (< 0.05) indicating a strong correlation between ET_o,s_ and ET_o,i_. Among 13 empirical models, ET_o,5_ recognized as the worst model with a lower accuracy and reliability than other models as this model produced the r-value of 0.78 which is less than 0.80. From the comparisons of 13 empirical models by RE, RMSE, MAE, NSE, MBE and scatter plots, ET_o,6_ model found as the best suitable alternative model against the ET_o,s_ model for calculating ETo for all the sub-regions and whole Bangladesh.

### Exploring the most suitable model for ET_o_ computation using the RF algorithm

The importance degree analysis of the empirical models was calculated by the RF method at different sub-regions of Bangladesh. The RF model depicted a suitable significant model followed by other analyses against the FAO56-PM model for all of the sub-regions (Table 6). The best performed model is highlighted by bold face and the least performed model is marked by italic face (Table 6) for estimating ETo. As can be seen from Table 6, the ET_o,6_ outperformed for ETo estimation in all the sub-regions of Bangladesh as it produced a comparatively higher importance degree. Whereas, ET_o,5_ found as the worst model producing the lowest importance degree among all the models.

**Table 6:** Importance degree of 13 empirical models against FAO56-PM model in seven sub-regions in Bangladesh using RF model.

Models	I	II	III	IV	V	VI	VII
ET_o,1_	4.27	6.27	9.03	2.22	2.46	5.10	5.07
ET_o,2_	3.99	6.16	4.34	2.34	5.81	5.81	4.87
ET_o,3_	9.02	7.27	5.61	2.51	5.15	5.15	4.87
ET_o,4_	7.46	6.57	7.53	5.00	9.47	2.71	4.45
ET_o,5_	*3.78*	*2.69*	*3.57*	*2.12*	*2.71*	*2.46*	*4.14*
ET_o,6_	**16.00**	**24.65**	**26.70**	**23.57**	**28.23**	**28.23**	**19.61**
ET_o,7_	8.70	8.14	7.01	7.68	5.10	9.47	8.65
ET_o,8_	9.38	7.99	7.48	9.98	9.55	9.55	8.91
ET_o,9_	8.37	8.43	5.40	8.51	6.00	6.00	6.11
ET_o,10_	8.36	8.74	7.95	8.30	7.78	7.78	6.96
ET_o,11_	8.81	6.48	7.05	11.05	8.98	8.98	8.89
ET_o,12_	7.90	3.85	4.84	10.30	2.91	2.91	10.90
ET_o,13_	3.97	2.76	3.46	6.44	5.85	5.85	6.58

Bold face indicates the highest importance degree while italic face denotes the least importance degree.

### Validation of the best alternative model for ET_o,s_

Validation is very important for determining the most suitable model from several potential models. All the applied empirical models need to validate to ensure whether the pre-analyses gave the accurate results or not and to find the best suitable alternative empirical model against the FAO56-PM. Validation was undertaken by utilizing the linear correlation method. Following Peng et al.^11^, the linear correlation was calculated by the below Eq. (33):

33 $${ET}_{o,s}=\frac{{ET}_{o,i}-b}{a}$$

where, *a* and *b* denoted as fitted coefficients.

Table 7 shows the fitted *a, b* and R^2^ values of correlation between ET_o,s_ and ET_o,i_ in seven sub-regions and whole Bangladesh. A strong correlation between ET_o,s_ and ET_o,i_ was found in all sub-regions and whole Bangladesh. Values of R^2^ is greater than 0.8 in every sub-regions and in whole Bangladesh for all the 13 empirical models indicating a strong correlation between ET_o,s_ and ET_o,i_. The model which is highly correlated with the ET_o,s_ is highlighted with light pink color. Among 13 models ET_o,6_ performed best, producing greater R^2^ values than the other models. ET_o,6_ model is simple among all the models used in this study as this model utilized only Tmax and Rs. Rs is calculated from T_max_ and T_min_. T_max_ and T_min_ are available everywhere in each region and can estimate easily. So, it can be affirmed that the ET_o,6_ is the best suitable, preferred, accurate, simple and reliable model, highly consisted with the results of pre-analyses (spatial and temporal distribution, performance evaluation and importance degree analysis), for ETo estimation in all the sub-regions and whole Bangladesh.

**Table 7:** Fitted *a, b* and R^2^ values of correlation between ET_o,s_ and ET_o,i_ in seven sub-regions and whole Bangladesh.

Sub-region	Parameter	ET_0,1_	ET_0,2_	ET_0,3_	ET_0,4_	ET_0,5_	ET_0,6_	ET_0,7_	ET_0,8_	ET_0,9_	ET_0,10_	ET_0,11_	ET_0,12_	ET_0,13_
I	A	0.738	0.761	0.435	0.645	0.058	1.089	0.712	0.697	0.760	1.151	1.479	0.683	0.089
	B	0.954	1.229	1.559	0.815	0.045	− 0.312	1.292	0.619	0.196	0.215	− 0.458	0.879	0.078
	R^2^	0.901	0.919	0.911	0.978	0.917	0.989	0.988	0.986	0.984	0.986	0.984	0.988	0.921
II	A	0.781	0.794	0.787	0.688	0.055	1.076	0.694	0.702	0.754	1.038	1.393	0.641	0.078
	B	0.934	1.310	1.222	0.782	0.040	− 0.259	1.335	0.608	0.225	0.155	− 0.188	1.003	0.045
	R^2^	0.958	0.96	0.942	0.956	0.86	0.982	0.98	0.982	0.98	0.974	0.978	0.98	0.88
III	A	0.744	0.782	0.752	0.651	0.054	1.032	0.695	0.667	0.716	1.051	1.38	0.656	0.067
	B	1.061	1.333	1.342	0.908	0.045	− 0.049	1.351	0.760	0.395	0.163	− 0.070	0.956	0.055
	R^2^	0.956	0.96	0.961	0.955	0.848	0.988	0.976	0.987	0.974	0.974	0.974	0.974	0.854
IV	A	0.713	0.747	0.735	0.624	0.060	1.051	0.694	0.675	0.727	1.051	1.401	0.660	0.079
	B	1.154	1.435	1.255	0.990	0.050	− 0.162	1.306	0.660	0.279	0.062	− 0.236	0.894	0.058
	R^2^	0.943	0.951	0.964	0.941	0.856	0.984	0.978	0.978	0.978	0.976	0.976	0.978	0.861
V	A	0.782	0.805	0.741	0.687	0.071	1.000	0.631	0.591	0.644	0.953	1.302	0.582	0.085
	B	1.006	1.315	1.233	0.856	0.097	0.099	1.564	1.012	0.628	0.457	0.179	1.179	0.099
	R^2^	0.956	0.962	0.967	0.955	0.803	0.974	0.972	0.970	0.970	0.970	0.972	0.968	0.812
VI	A	0.619	0.643	0.687	0.543	0.083	0.817	0.520	0.493	0.538	0.813	1.084	0.488	0.098
	B	1.504	1.819	1.612	1.295	0.115	0.566	1.868	1.233	0.866	0.833	0.751	1.424	0.122
	R^2^	0.955	0.956	0.954	0.952	0.866	0.978	0.964	0.966	0.966	0.968	0.970	0.964	0.871
VII	A	0.744	0.799	0.778	0.649	0.052	0.995	0.67	0.626	0.663	1.023	1.363	0.646	0.056
	B	1.091	1.306	1.124	0.943	0.033	0.097	1.455	0.882	0.493	0.107	0.009	0.994	0.043
	R^2^	0.968	0.972	0.952	0.966	0.914	0.979	0.98	0.976	0.974	0.976	0.974	0.98	0.911
Whole BD	A	0.796	0.846	0.784	0.696	0.061	1.046	0.654	0.691	0.706	1.098	1.418	0.660	0.071
	B	0.818	1.054	1.102	0.699	0.056	− 0.151	0.763	1.348	0.363	− 0.091	− 0.258	0.929	0.057
	R^2^	0.743	0.766	0.787	0.736	0.615	0.856	0.803	0.849	0.811	0.843	0.851	0.825	0.601

## Discussions

Increasing and decreasing as well as significant and non-significant trends were found across the country from 1980 to 2017. The rates between increasing and decreasing of ET_o,s_ was 72.18 to − 72.17 mm per decade in this study. For example, Bhola, Cumilla, Feni, Rangamati and Patuakhali showed a significant increasing trend of ET_o,s_ and non-significant decreasing trend of ET_o,s_ found in Faridpur, Madaripur, Dhaka and Barishal of sub-regions I and VII (southeastern and south-central regions). The significant decreasing trend of ET_o,s_ showed by the sub-regions of II, III, IV and V. Cox’s Bazar, Teknaf, Sandwip, Chattogram, Jashore, Mymensingh and Khulna showed a significant decreasing trend of ET_o,s_. From the above results, it is evident that the trend of ET_o,s_ was decreasing gradually in Bangladesh from 1980 to 2017. Rahman et al.^15^ found that most of the area of Bangladesh showed decreasing trends and some parts of the study area showed an increasing trend of ET_o,s_ which is analogous to the results of this study. Decreasing the trend of ET_o,s_ may be the results of worldwide climate change impacts. Spatial distribution of the long-term mean monthly between ET_o,s_ and ET_o,6_ (Abtew) in Bangladesh shown a homogenous pattern. By contrary, ET_o,5_ (WMO) and ET_o,13_ (Turc) models produced the least close values to ET_o,s_ in terms of spatial distribution. Temporal distribution of long-term monthly ET_o,s_, and ET_o,i_ in Bangladesh found that the highest and lowest rate of ETo occurred in April and January–December, respectively. Peng et al.^11^ and Li et al.^24^ showed that the highest and lowest rate of ET_o,s_ occurred in China in July (dissimilar to this study) and January–December which is similar to this study. Long term inter-annual variation of ET_o,s_, and ET_o,i_ in Bangladesh revealed that ET_o,5_ and ET_o,13_ models produced very lower values, identifying the least performed method for calculating ETo. Previous studies^5,11,24,29^ along with this study revealed that ETo values obtained by both ET_o,s_ and ET_o,i_ models were very closer to each other in the month of January–December (cold season) and highest discrepancy occurred among them in the hot summer season.

Long term monthly RE (relative error) of ET_o,i_ revealed that the WMO and Turc models were the least suitable with greater RE values and Abtew model was an optimal alternative with lower RE values against the FAO56-PM for calculating ETo in Bangladesh. The values of RMSE, MAE, NSE, and MBE had the strong concurrence with the RE exploring the same results as Abtew (ET_o,6_) was the best alternative and WMO (ET_o,5_); Turc (ET_o,13_) were the least suitable model for estimating ETo in Bangladesh. Gabriela and Irmak^73^ evaluated the impact of the meteorological variables on the estimates of 13 empirical models in various regions and found that the Doorenbos and Pruitt (ET_o,12_) ranked top in the three regions of Iran under sub-humid to sub-arid climate conditions which are in disagreement with the results of this study. Identification of the best suitable model against ET_o,s_ varies from region to region and country to country. It might be due to the variation of geographical and meteorological variations from one country to another country and input model combinations. Correlation between long term daily ET_o,s_ and ET_o,i_ in Bangladesh was explored that a very strong correlation aligns with no one line (1:1) existed between ET_o,s_ and ET_o,6_ (Abtew) with the r values of 0.92. Li et al.^24^ also found a strong correlation (R^2^ was 0.972) between daily ET_o,s_ and ET_o,13_ (Valiantzas 3) in China. Xystrakis and Matzarakis^35^ found a strong correlation (r-value 0.993) between monthly ET_o,s_, and ET_o,i_ (Turc) in Greece. There also existed a strong correlation (r-value 0.996) between ET_o,s_, and ET_o,i_ (Blaney–Criddle) explored by Tabari et al.^40^ in Iran. Peng et al.^11^ found the largest correlation between monthly ET_o,s_, and ET_o,6_^53^ in China. Present study found the Abtew (ET_o,6_) model as the most reliable model for estimating ETo, compared to the other empirical models in all the sub-regions of Bangladesh, as this model produced a higher importance degree.

Evaluation of the performance of different empirical models for estimating the ET_o,s_ was finally validated by Eq. (33). This study explored that Abtew (ET_o,6_) model outperformed other models with R^2^ values ranged from 0.856 to 0.989 at all the sub-regions of Bangladesh. This is in good agreement with the earlier performance appraisal results in which RMSE, MAE, MBE, and NSE are the lowest in ET_o,6_ model. The main reason is that this model has high precision, easy, consistent; requiring less climatic datasets and strong association with FAO56-PM. This model provides satisfactory outcomes and generally uses simple computable parameters and has easy model forms. Djaman et al.^33^ also found a similar result as this study that the Abtew was the best alternative model against the FAO56-PM in New Mexico, USA. Li et al.^24^ found Valiantzas 3 as the outperformed model among 13 empirical models for estimating ETo with R^2^ values ranged from 0.882 to 0.993. Peng et al.^11^ explored ET_o,6_^56^ as the best-performed model at all the sub-regions and EMC among ten empirical models for calculating ETo with R^2^ values ranged from 0.87 to 0.99. Shiri^5^ showed Priestley–Taylor outweighed the other 6 empirical models for estimating ETo with R^2^ values ranged from 0.636 to 0.792. Mohawesh^38^ found Penman as the best-performed model in different regions of Jordan for estimating ET_o,s_ with R^2^ values ranged from 0.66 to 0.78^74^. Similarly, the mass-transfer-based model was the optimal model in computing ETo compared to the other models in humid regions in Iran^40^ and forest regions in Greece^32^. Based on the accuracy, reliability, simplicity and higher correlation with ET_o,s_, the most suitable method for ETo calculation in Bangladesh is ET_o,6_ model.

## Conclusions

In this study, daily meteorological datasets from 20 weather stations from seven sub-regions in Bangladesh for the period of 1980–2017 were used. A widespread comparison between ET_o,s_ (calculated by FAO56-PM) and ET_o,i_ (calculated by HS, HM1, HM2, BT, WMO, ABT, IR1, IR2, MAK, PT, JH, TAB, TR, respectively) has been carried out. The possible roles of these 13 empirical models against the FAO56-PM were also explored in this study. Out of 20 stations, 5 stations showed an increasing trend of ET_o,s_; 11 stations showed a decreasing trend and 4 stations showed no trend of ET_o,s_. Spatiotemporal distribution of ET_o,s_, and ET_o,i_ revealed that the model proposed by Abtew model showing the closest distribution of ET_o,i_ to ET_o,s_. RE, RMSE, MAE, MBE, and NSE were employed for evaluating the empirical models which were identified ET_o,6_ as the outperformed model with the lowest errors for calculating ETo in different sub-regions and whole Bangladesh. By contrast, ET_o,5_ (WMO) and ET_o,13_ (Turc) models selected as the poorer alternative models with the higher statistical errors. RF model also confirmed the Abtew as the outperformed model. The linear regression model showed that a strong linear correlation was found between FAO56-PM and Abtew model. Validation by using Eq. (33) explored the similar outcomes that the ET_o,6_ model outperformed than the other models. This study recommends the model proposed by the Abtew (ET_o,6_) as the best alternative model with high accuracy, reliability and lowest errors for all the sub-regions and whole Bangladesh for calculating ETo when full climatic datasets for FAO56-PM model are unavailable. Future study should be focused on the evaluation of machine learning ensemble models for estimating daily ETo in Bangladesh. This research is a vital scientific contribution to ETo quantification and influential empirical models in Bangladesh where the large set of meteorological datasets could not be acquired. This study provides an important guidance for agricultural water practices, hydrological processes and irrigation management in Bangladesh, also useful as well as the similar subtropical climate region elsewhere in the world.

## Supplementary information

Supplementary Information.

## Data Availability

The datasets investigated in the present research are easily reached from the corresponding author on request.
