# Supplementary material for: The optimal alternative for quantifying reference evapotranspiration in climatic sub-regions of Bangladesh
Source: Sci Rep. 2020 Nov 19;10:20171. doi: 10.1038/s41598-020-77183-y (PMC7678841; doi:10.1038/s41598-020-77183-y)
Supplement: Supplementary file 1 — Supplementary Information. [file 41598_2020_77183_MOESM1_ESM.docx]

**The optimal alternative for quantifying reference evapotranspiration in climatic sub-regions of Bangladesh**

Roquia Salam^1^, Abu Reza Md. Towfiqul Islam^1^, Quoc Bao Pham^2,3^, Majid Dehghani^4^, Nadhir Al-Ansari^5^, Nguyen Thi Thuy Linh^6,7,*^

^1^Department of Disaster Management, Begum Rokeya University, Rangpur 5400, Bangladesh

^2^Environmental Quality, Atmospheric Science and Climate Change Research Group, Ton Duc

Thang University, Ho Chi Minh City, Vietnam

^3^Faculty of Environment and Labour Safety, Ton Duc Thang University, Ho Chi Minh City,

Vietnam

^4^ Civil Engineering Department, Vali-e-Asr University of Rafsanjan, Rafsanjan, Iran

^5^Department of Civil, Environmental and Natural Resources Engineering, Lulea University of Technology, 97187, Lulea, Sweden

^6^Institute of Research and Development, Duy Tan University, Danang 550000, Vietnam

^7^Faculty of Environmental and Chemical Engineering, Duy Tan University, Danang 550000, Vietnam

*Corresponding authors: nguyentthuylinh58@duytan.edu.vn

**Supplementary materials**

**List of supplementary Figures**


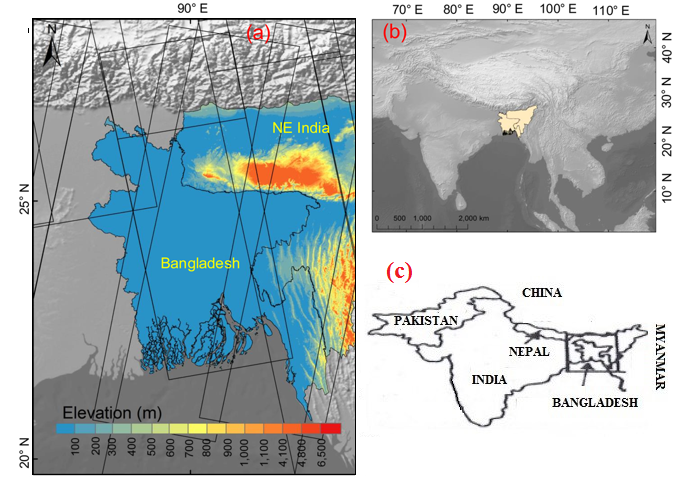
Figure S1. Topography and geographic setting of Bangladesh (Map Source: Singha et al**^75^**) showing the location of the study area.
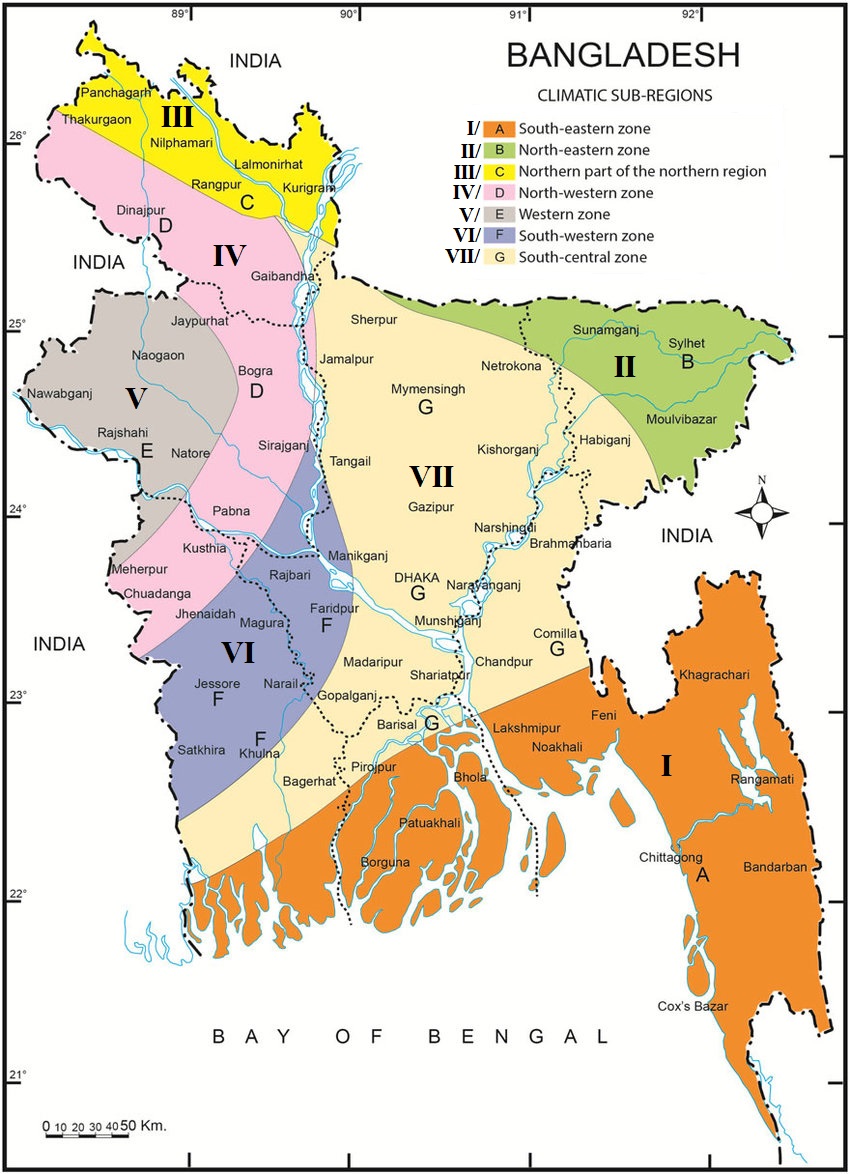


**Figure S2.** Climatic sub-regions of Bangladesh; Modified after Banglapedia 2014 ([***www.en***.banglapedia.org](https://www.google.com/search?q=www.en.banglapedia.org&tbm=isch&nfpr=1&bih=568&biw=1366&rlz=1C1JZAP_enBD911BD911&hl=en&sa=X&ved=2ahUKEwj2rZKztMXsAhW_K7cAHRHOCUkQvgV6BAgBEBQ)), by ArcGis 10.5 ([www.esri.com](http://www.esri.com)).

**List of supplementary Tables**

**Table S1.** Short review of the empirical models for the estimation of reference evapotranspiration (2011-2019).

| **Authors** | **Empirical models** | | **Input variables** | **Region** | **Performance metrics** | **Best performed model** |
| --- | --- | --- | --- | --- | --- | --- |
| Farzanpour et al**^29^** | Temperature-based (BC, HS1, HS2, HS3, HS4, SD); Radiation-based (IR, JH, RC, TAB1, TAB2); Mass transfer-based (DT, TB, MEY, WMO, MG, PM, AH, BW, RW) | Tave, Tmin, Tmax, U2, Hr, Ra, Rs, ea, es | | Iran | SI, MAE | Blaney–Criddle and Schendel |
| Djaman et al**^33^** | Temperature-based (BT, DJ, RK, AD1, AD2 , AL, HS); Radiation based (CP, HV, IR, TV, DA, JH, TAB1, TAB2, ABT1, ABT2, ABT3, MAK, CH); Combined (VA1, VA2, VA3, VA4, VA5); Mass transfer based (AH, BW, DT, MG, MEY, PM, TB, WMO, RW) | Tave, Tmin, Tmax, U2, Hr, Ra, Rs, ea, es | | New Mexico, USA | MBE, MAE, RMSE, PE, R^2^ | Valiantzas, Makkink,  Calibrated Hargreaves, Abtew, Jensen-Haise, and Caprio |
| Muhammad et al**^34^** | Temperature-based (IV, HM, PD, SD, FAO BC, LC, KR, HV, TV, RZ); Radiation-based (MAK, TR, JS, PT, MG, CP, JN, ABT, IR1, IR2); Mass transfer-based (DT, TB, MEY, RW, PM, AH, BP, WMO, MG, SZ); Combination-based (FAO PM). | Tave, Tmin, Tmax, U2, Hr, Ra, Rs, Rn, ea, es | | Peninsular Malaysia | NRMSE, %BIAS, md, KGE | FAO Penman-Monteith, Priestley-Taylor, Ivanov, Dalton and Meyer. |
| Bourletsikas et al**^32^** | Mass-transfer based (AH, MG, PM, RK, WMO); Combined (CO, STU, VA1, VA2); Radiation-based (ABT, CP, DBK, FAO24R, HA, JH, MAK, MGB, PT, TR, HV, HS, HSM1, HSM2, THM) | Tave, Tmin, Tmax, U2, Hr, Ra, Rs, Rn, ea, es | | Greece | AAE, SD, R^2^, RMSE, MV, MBE, MAE, EF, WR^2^, d, rt, rMSE, rMAE. | Copais, Hargreaves and Valiantzas2 |
| Li et al**^24^** | Temperature-based (HS); Mass transfer-based (PM, WMO, TB); Radiation-based (MAK, PT, JH, ABT, IR, TAB); Combination-based (VA1, VA2, VA3) | Tave,  Tmin, Tmax, U2, Hr, Ra, Rs, Rn, ea, es | | China | RMSE, MAE, NSE | Valiantzas3 |
| Peng et al**^11^** | IR, MAK, PT, HS, DA, BT, DP, WRI, VA1, VA2. | Tave,  Tdew,  Tmin, Tmax, U2, Hr, Ra, Rs, Rn, ea, es | | China | RE, θ, NSE. | Berti |
| Tabari et al**^40^** | Pan evaporation-based (FAO24P, CC, AP, SN, MSN, PR, OR, FAO56P); Temperature-based (TH, BC, SD, HM1, HM2, HM3, HM4); Radiation-based (JH, MGB, RC, IR1, IR2, IR3); Mass transfer-based (DT, TB, MEY, RW, PM, AH, RK, BW, WMO, MG) | Tave, Tmin, Tmax, U2, Hr, Ra, Rs, P, ea, es | | Iran | RMSE, PE, MBE, R^2^ | Blaney–Criddle |
| Xystrakis and Matzarakis**^35^** | Temperature-Based (HV, MGB, RK, HM1, HM2, HM3, MC); Radiation-Based and Combination-based (HA, CP, JH, TR, MAK, DB) | Tave,  Tdew,  Tmin, Tmax, U2, Hr, Ra, Rs, Rn, ea, es, Cl. | | Greece | MSE, MAE, MBE, RMAE, RMSE | Hansen and  Turc |
| Mohawesh**^38^** | PM, HV, HM1, HM2, FAO24R, PT, MAK, FAO24P. | Tmin, Tmax, U2, Hr, Ra, Rs, Rn, ET | | Jordan | MBE, MAE, RMSE | Penman |

Note: Blaney & Criddle=BC, Hargreaves-Samani=HS, Schendel=SD, Irmak=IR, Jensen-Haise=JH, Ritchie=RC, Tabari=TAB, Dalton=DT, Trabert=TB, Meyer=MEY, Mahringer=MG, Penman=PM, Albrecht=AH, Brockamp & Wenner=BW, Rohwer=RW, Berti=BT, Dorji=DJ, Romanenko= RK, Ahooghalandari= AD, Allen=AL, Caprio=CP, Hargreaves=HV, Trajkovic= TV, Droogers and Allen=DA, Abtew= ABT, Makkink=MAK, Calibrated Hargreaves= CH, Valiantzas= VA, Ivanov= IV, Hamon= HM, Papadakis= PD, Linacre= LC, Kharrufa= KR, Ravazzani= RZ, Turc= TR, Jensen= JS, Priestley-Taylor= PT, McGuinness= MG, Jones= JN, Brockamp= BP, Szasz= SZ, FAO Penman-Monteith= FAO PM, Solar Thermal Unit=STU, Copais=CO, De Bruin–Keijman= DBK, FAO24 Radiation=FAO24R, Hansen=HA, McGuinness–Bordne= MGB, Hargreaves–Samani Modified= HSM, Thornthwaite Modified= THM, Doorenbos-Pruitt= DP, Wright= WRI, Thornthwaite = TH, FAO-24 pan= FAO24P, Cuenca= CC, Allen and Pruitt= AP, Snyder=SN, Modified Snyder=MSN, Pereira=PR, Orang=OR, FAO-56 pan= FAO56P, Hargreaves Modified= HM, McCloud= MC, De Bruin= DB.

**Table S2.** The geographical location and daily average values of meteorological data for the selected stations in Bangladesh.

| **Stations** | **Latitude**  **(N)** | **Longitude**  **(E)** | **Altitude**  **(m)** | **Tmax**  **(^0^C)** | **Tmin**  **(^0^C)** | **Rs**  **(MJm^-2^d^-1^)** | **Ra**  **(MJm^-2^d^-1^)** | **U2**  **(ms^-1^)** | **Hr**  **(%)** | **ETo**  **(mmd^-1^)** |
| --- | --- | --- | --- | --- | --- | --- | --- | --- | --- | --- |
| **Bogura** | 24.85 | 89.37 | 17.90 | 29.91 | 21.04 | 16.69 | 32.84 | 1.06 | 78.14 | 3.69 |
| **Dhaka** | 23.77 | 90.38 | 8.45 | 30.15 | 21.93 | 17.00 | 33.10 | 1.12 | 74.40 | 3.75 |
| **Jashore** | 23.2 | 89.33 | 6.10 | 30.72 | 20.99 | 16.39 | 33.24 | 2.23 | 78.81 | 4.04 |
| **Mymensingh** | 24.73 | 90.42 | 18.00 | 29.78 | 20.95 | 16.04 | 32.87 | 1.03 | 79.77 | 3.46 |
| **Patuakhali** | 22.33 | 90.33 | 1.50 | 30.08 | 22.09 | 16.27 | 33.43 | 1.54 | 83.19 | 3.54 |
| **Rajshahi** | 24.37 | 88.7 | 19.50 | 30.11 | 20.56 | 17.25 | 32.97 | 1.00 | 78.18 | 3.78 |
| **Rangamati** | 22.37 | 92.15 | 68.89 | 29.82 | 21.00 | 17.35 | 33.43 | 0.71 | 79.01 | 3.62 |
| **Rangpur** | 25.73 | 89.27 | 32.61 | 28.96 | 20.25 | 16.60 | 32.63 | 1.03 | 80.26 | 3.53 |
| **Sylhet** | 24.9 | 91.88 | 33.53 | 29.06 | 20.62 | 16.48 | 33.32 | 1.07 | 78.89 | 3.54 |
| **Faridpur** | 23.6 | 89.85 | 8.10 | 30.14 | 21.42 | 17.27 | 33.14 | 1.17 | 79.01 | 3.72 |
| **Madaripur** | 23.17 | 90.18 | 7.00 | 30.82 | 21.45 | 16.51 | 33.25 | 0.78 | 78.86 | 3.71 |
| **Khulna** | 22.78 | 89.57 | 2.10 | 30.44 | 21.79 | 17.23 | 33.33 | 1.29 | 80.86 | 3.92 |
| **Barishal** | 22.72 | 90.37 | 2.10 | 29.88 | 21.39 | 16.97 | 33.35 | 1.33 | 83.78 | 3.55 |
| **Bhola** | 22.68 | 90.65 | 4.30 | 29.53 | 21.72 | 15.73 | 33.35 | 0.92 | 83.67 | 3.55 |
| **Chattogram** | 22.22 | 91.8 | 5.50 | 30.04 | 21.77 | 17.30 | 33.45 | 2.68 | 79.13 | 4.12 |
| **Cumilla** | 23.43 | 91.18 | 7.50 | 29.56 | 21.08 | 17.17 | 33.18 | 1.22 | 81.44 | 3.63 |
| **Cox’s Bazar** | 21.45 | 91.96 | 2.10 | 29.83 | 22.35 | 17.89 | 33.63 | 2.24 | 79.88 | 4.03 |
| **Feni** | 23.03 | 91.42 | 6.40 | 29.59 | 21.31 | 16.94 | 33.27 | 1.13 | 80.77 | 3.66 |
| **Sandwip** | 22.48 | 91.43 | 2.10 | 29.97 | 22.02 | 17.37 | 33.41 | 1.27 | 83.41 | 4.07 |
| **Teknaf** | 20.87 | 92.3 | 5.00 | 30.38 | 22.17 | 17.91 | 33.75 | 1.51 | 79.14 | 3.65 |

**Table S3.** Detailed information of the missing data management of daily maximum temperature, minimum temperature, relative humidity and wind speed of 20 stations over Bangladesh.

| **Stations** | **Year** | **Number of missing days** | | | | **Neighbor station from where missing data filled-up** |
| --- | --- | --- | --- | --- | --- | --- |
|  |  | **Tmax** | **Tmin** | **Hr** | **U2** |  |
| **Bogura** | 1980 | 3 | - | - | - | Rajshahi |
|  | 1981 | 2 | - | - | - |  |
|  | 1982 | 9 | 4 | - | - |  |
|  | 1983 | 2 | - | - | - |  |
|  | 1985 | 2 | - | - | - |  |
|  | 1986 | 1 | - | - | - |  |
|  | 1987 | 12 | 8 | 6 | 5 |  |
|  | 1989 | 2 | - | - | - |  |
|  | 1994 | 1 | - | - | - |  |
|  | 1995 | 2 | - | - | - |  |
|  | 1997 | 1 | - | - | - |  |
|  | 1998 | 1 | - | - | - |  |
|  | 2004 | 16 | - | - | - |  |
|  | 2013 | 30 | 30 | - | - |  |
| **Dhaka** | 1980 | 2 | 2 | - | 2 | Tangail, Mymensingh |
|  | 1981 | 8 | 3 | - | - |  |
|  | 1982 | 4 | 2 | - | - |  |
|  | 1983 | 4 | 3 | 4 | - |  |
|  | 1985 | 1 | - | - | - |  |
|  | 1986 | 1 | - | - | - |  |
|  | 1987 | - | 2 | 2 | 1 |  |
|  | 1988 | 1 | - | - | - |  |
|  | 1989 | 1 | 1 | - | - |  |
|  | 1992 | 1 | - | - | - |  |
|  | 1995 | 1 | - | - | - |  |
|  | 2008 | 1 | - | - | - |  |
| **Jashore** | 1980 | 14 | 6 | - | - | Khulna |
|  | 1982 | 3 | 1 | - | - |  |
|  | 1984 | 2 | - | - | - |  |
|  | 1985 | 3 | 10 | - | - |  |
|  | 1986 | 4 | 43 | - | - |  |
|  | 1987 | 1 | - | - | - |  |
|  | 1988 | 1 | - | - | - |  |
|  | 1989 | 1 | - | - | - |  |
|  | 1991 | 5 | 7 | - | - |  |
|  | 1992 | 3 | - | - | - |  |
|  | 1993 | 2 | - | - | - |  |
|  | 1994 | 1 | 1 | - | - |  |
|  | 1995 | 1 | - | - | - |  |
|  | 1996 | 1 | - | - | - |  |
|  | 1997 | - | 1 | - | - |  |
|  | 1998 | 3 | - | - | - |  |
|  | 1999 | 1 | - | - | - |  |
|  | 2000 | 2 | - | - | - |  |
|  | 2002 | 2 | - | - | - |  |
|  | 2004 | 2 | 1 | - | - |  |
| **Mymensingh** | 1980 | - | 5 | - | - | Tangail, Dhaka |
|  | 1981 | 1 | 365 | - | - |  |
|  | 1982 | 1 | 365 | - | - |  |
|  | 1984 | 2 | 120 | - | - |  |
|  | 1985 | 4 | 20 | - | - |  |
|  | 1987 | - | 3 | - | - |  |
|  | 1991 | - | 1 | - | - |  |
|  | 1994 | 2 | - | - | - |  |
|  | 1995 | 1 | - | - | - |  |
|  | 1996 | 1 | - | - | - |  |
|  | 1998 | 3 | - | - | - |  |
| **Patuakhali** | 1980 | 365 | 365 | 365 | 365 | Barishal, Bhola |
|  | 1981 | 2 | 2 | 2 | 2 |  |
|  | 1983 | 2 | 3 | 2 | - |  |
|  | 1986 | 1 | 3 | - | - |  |
|  | 1987 | 22 | 7 | - | - |  |
|  | 1990 | 1 | - | - | - |  |
|  | 1993 | - | 1 | - | - |  |
|  | 1997 | 5 | 2 | - | - |  |
|  | 1998 | 1 | - | - | - |  |
|  | 1999 | 1 | - | - | - |  |
|  | 2003 | 1 | 1 | - | - |  |
| **Rajshahi** | 1980 | 5 | 2 | - | - | Bogura, Ishurdi |
|  | 1981 | 113 | 12 | 11 | - |  |
|  | 1982 | 150 | 7 | - | - |  |
|  | 1983 | 1 | 2 | - | - |  |
|  | 1984 | 1 | - | - | - |  |
|  | 1985 | 8 | 6 | - | 6 |  |
|  | 1987 | 7 | 7 | 6 | 6 |  |
|  | 1988 | 3 | 2 | 2 | 2 |  |
|  | 1989 | 1 | - | - | - |  |
|  | 1990 | 1 | - | - | - |  |
|  | 1991 | 2 | - | - | - |  |
|  | 1994 | 4 | - | - | - |  |
|  | 1995 | 3 | - | - | - |  |
|  | 1996 | 1 | - | - | - |  |
|  | 1997 | 3 | 13 | - | - |  |
|  | 1998 | 2 | - | - | - |  |
|  | 1999 | 3 | - | - | - |  |
|  | 2003 | 1 | - | - | - |  |
| **Rangamati** | 1980 | 16 | 3 | 3 | 31 | Chattogram |
|  | 1981 | 91 | 91 | 91 | 206 |  |
|  | 1983 | 2 | - | - | - |  |
|  | 1984 | 1 | - | - | - |  |
|  | 1985 | 1 | - | - | - |  |
|  | 1987 | 6 | 6 | 5 | 5 |  |
|  | 1991 | 2 | 1 | - | - |  |
|  | 1993 | - | 87 | - | - |  |
|  | 1994 | - | 17 | - | - |  |
|  | 1995 | 1 | - | - | - |  |
|  | 1996 | 1 | - | - | - |  |
|  | 1998 | 1 | - | - | - |  |
|  | 1999 | 1 | - | - | - |  |
|  | 2001 | 1 | - | - | - |  |
|  | 2005 | 2 | - | - | - |  |
|  | 2007 | 1 | - | - | - |  |
|  | 2013 | 1 | 1 | - | - |  |
|  | 2015 | - | 59 | 59 | - |  |
| **Rangpur** | 1980 | 24 | 3 |  | - | Dinajpur, Sydpur |
|  | 1981 | 112 | 104 | 92 | 89 |  |
|  | 1983 | 5 | 3 | 2 | 2 |  |
|  | 1984 | 2 | - | - | - |  |
|  | 1986 | - | 1 | - | - |  |
|  | 1993 | 1 | - | - | - |  |
|  | 1994 | 1 | - | 13 | - |  |
|  | 1995 | 2 | - | - | - |  |
|  | 1996 | 1 | - | - | - |  |
|  | 1997 | 1 | - | - | - |  |
|  | 2004 | 1 | - | - | - |  |
|  | 2006 | 3 | 3 | - | - |  |
| **Sylhet** | 1980 | 5 | 5 | - | - | Srimangal |
|  | 1983 | 1 | - | - | - |  |
|  | 1987 | 15 | 285 | 6 | 6 |  |
|  | 1990 | 1 | - | - | - |  |
|  | 1995 | 6 | 2 | - | - |  |
|  | 1998 | 1 | - | - | - |  |
| **Faridpur** | 1980 | 4 | - | - | - | Madaripur |
|  | 1981 | 3 | 3 | 3 | 3 |  |
|  | 1982 | 6 | 1 | - | - |  |
|  | 1983 | 6 | 6 | - | - |  |
|  | 1984 | 1 | - | - | - |  |
|  | 1985 | 1 | - | - | - |  |
|  | 1988 | 1 | - | - | - |  |
|  | 1991 | 1 | - | - | - |  |
|  | 1994 | 2 | - | - | - |  |
|  | 1995 | 1 | 2 | - | - |  |
|  | 1996 | 4 | 1 | - | - |  |
|  | 2000 | 1 | - | - | - |  |
|  | 2004 | - | 13 | - | - |  |
|  | 2007 | 1 | - | - | - |  |
| **Madaripur** | 1980 | 7 | 1 | - | - | Faridpur |
|  | 1981 | 153 | 153 | 153 | 153 |  |
|  | 1983 | 1 | - | - | - |  |
|  | 1984 | 2 | 2 | 2 | - |  |
|  | 1986 | 1 | 1 | - | - |  |
|  | 1987 | 1 | 195 | - | - |  |
|  | 1988 | 6 | 184 | 6 | - |  |
|  | 1989 | 1 | 58 | - | - |  |
|  | 1990 | - | 60 | - | - |  |
|  | 1995 | 1 | - | - | - |  |
|  | 1996 | - | 8 | - | - |  |
|  | 1997 | 1 | - | - | - |  |
|  | 1998 | 3 | 3 | 2 | - |  |
|  | 1999 | 159 | - | - | - |  |
|  | 2001 | 1 | - | - | - |  |
|  | 2007 | - | 8 | - | - |  |
|  | 2013 | - | 40 | - | - |  |
| **Khulna** | 1980 | 15 | 9 | - | - | Jashore, Satkhira |
|  | 1982 | 3 | 3 | - | - |  |
|  | 1983 | 31 | 31 | 31 | 31 |  |
|  | 1984 | 1 | - | - | - |  |
|  | 1985 | 1 | - | - | - |  |
|  | 1986 | 1 | - | - | - |  |
|  | 1988 | - | 1 | - | - |  |
|  | 1989 | 1 | - | - | - |  |
|  | 1990 | 1 | - | - | - |  |
|  | 1994 | 1 | - | - | - |  |
|  | 1995 | 2 | - | - | - |  |
|  | 1996 | 1 | - | - | - |  |
|  | 1997 | 1 | - | - | - |  |
|  | 1999 | 1 | 2 | - | - |  |
|  | 2005 | - | 1 | - | - |  |
| **Barishal** | 1980 | 8 | 3 | - | - | Patuakhali, Bhola |
|  | 1981 | 1 | 1 | - | - |  |
|  | 1982 | 1 | 1 | - | - |  |
|  | 1983 | 2 | - | - | - |  |
|  | 1986 | 2 | - | - | - |  |
|  | 1987 | 6 | 6 | 6 | 6 |  |
|  | 1992 | 1 | - | - | - |  |
|  | 1994 | - | 2 | - | - |  |
|  | 1995 | 3 | 3 | - | - |  |
|  | 1996 | 1 | - | - | - |  |
|  | 1998 | 2 | 1 | - | - |  |
|  | 1999 | 1 | - | - | - |  |
| **Bhola** | 1980 | 6 | 1 | - | - | Patuakhali,  Sandwip |
|  | 1981 | 2 | - | - | - |  |
|  | 1984 | 2 | - | - | - |  |
|  | 1987 | 6 | 6 | 5 | 5 |  |
|  | 1991 | 1 | 1 | - | - |  |
|  | 1992 | 16 | 16 | 14 | - |  |
|  | 1994 | 4 | 2 | 2 | 2 |  |
|  | 1995 | 1 | - | - | - |  |
|  | 1996 | 3 | 3 | - | - |  |
|  | 1997 | - | 1 | - | - |  |
|  | 2002 | 199 | - | - | - |  |
|  | 2004 | 3 | 8 | - | - |  |
| **Chattogram** | 1980 | 4 | 7 | - | - | Rangamati |
|  | 1981 | 39 | 33 | 32 | - |  |
|  | 1982 | 1 | 1 | - | - |  |
|  | 1983 | 3 | 2 | - | - |  |
|  | 1984 | - | 1 | - | - |  |
|  | 1985 | - | 1 | - | - |  |
|  | 1986 | 3 | - | - | - |  |
|  | 1987 | 14 | 7 | 6 | - |  |
|  | 1988 | - | 2 | - | - |  |
|  | 1991 | 44 | 45 | 40 | 40 |  |
|  | 1993 | - | 1 | - | - |  |
|  | 1995 | 1 | - | - | - |  |
|  | 1998 | 1 | - | - | - |  |
|  | 2001 | 5 | 1 | - | - |  |
|  | 2013 | - | 1 | - | - |  |
| **Cumilla** | 1980 | 4 | 2 | - | - | Feni, Dhaka |
|  | 1981 | 6 | 5 | - | 1 |  |
|  | 1982 | - | 1 | - | - |  |
|  | 1983 | 47 | 7 | - | - |  |
|  | 1984 | 2 | 2 | 2 | 2 |  |
|  | 1987 | 7 | 7 | 7 | 7 |  |
|  | 1990 | 2 | 1 | - | - |  |
|  | 1992 | 6 | 6 | - | - |  |
|  | 1993 | 1 | - | - | - |  |
|  | 1995 | 4 | 3 | - | - |  |
| **Cox’s Bazar** | 1980 | 67 | 68 | 61 | 61 | Teknaf |
|  | 1981 | 6 | 5 | - | - |  |
|  | 1982 | 3 | 2 | - | - |  |
|  | 1983 | 3 | - | - | - |  |
|  | 1984 | 26 | 7 | - | - |  |
|  | 1985 | 44 | 37 | - | - |  |
|  | 1986 | 21 | 21 | - | - |  |
|  | 1987 | 15 | 13 | 5 | 5 |  |
|  | 1988 | 8 | 3 | - | - |  |
|  | 1991 | 9 | 4 | - | - |  |
|  | 1992 | 1 | - | - | - |  |
|  | 1993 | 1 | - | - | - |  |
|  | 1995 | 7 | 3 | - | - |  |
|  | 1998 | 3 | - | - | - |  |
|  | 2000 | 1 | - | - | - |  |
| **Feni** | 1980 | 11 | 6 | - | - | Cumilla |
|  | 1981 | 6 | 2 | 3 | 2 |  |
|  | 1982 | 4 | 1 | - | - |  |
|  | 1983 | 33 | 32 | 31 | 31 |  |
|  | 1984 | 7 | - | - | - |  |
|  | 1985 | 5 | 2 | - | - |  |
|  | 1987 | 6 | 5 | 5 | 5 |  |
|  | 1989 | 1 | 1 | - | - |  |
|  | 1991 | - | 1 | - | - |  |
|  | 1994 | 1 | - | - | - |  |
|  | 1998 | - | - | - | 1 |  |
|  | 2001 | 1 | - | - | - |  |
|  | 2003 | 62 | 27 | 8 | - |  |
|  | 2005 | 31 | 31 | 31 | 31 |  |
| **Sandwip** | 1980 | 5 | 4 | - | - | Bhola |
|  | 1982 | 3 | - | - | - |  |
|  | 1983 | 2 | - | - | - |  |
|  | 1984 | 5 | - | - | - |  |
|  | 1985 | 3 | 2 | - | - |  |
|  | 1987 | 7 | 7 | 7 | 7 |  |
|  | 1991 | 39 | 39 | 37 | 37 |  |
|  | 1994 | 1 | 2 | - | - |  |
|  | 1996 | - | 22 | - | - |  |
|  | 1998 | 1 | 1 | - | - |  |
|  | 2000 | 146 | 147 | - | - |  |
|  | 2001 | 16 | 9 | - | - |  |
|  | 2002 | 365 | 365 | 122 | 122 |  |
|  | 2003 | 365 | 365 | 365 | 365 |  |
|  | 2004 | 260 | 260 | 31 | 31 |  |
|  | 2012 | 31 | 31 | 31 | - |  |
| **Teknaf** | 1980 | 4 | 4 | - | - | Cox’s Bazar |
|  | 1981 | 1 | - | - | - |  |
|  | 1982 | 3 | 2 | 2 | 2 |  |
|  | 1983 | 60 | 31 | 31 | 31 |  |
|  | 1984 | 93 | - | - | - |  |
|  | 1987 | 8 | 7 | 7 | 7 |  |
|  | 1991 | 5 | 13 | - | - |  |
|  | 1994 | 28 | 150 | 26 | - |  |
|  | 1995 | - | 3 | - | - |  |
|  | 1998 | 1 | - | - | - |  |
|  | 2006 | - | - | - | 1 |  |
|  | 2009 | 1 | - | - | - |  |
